# Chemoselective Preparation of New Families of Phenolic-Organoselenium Hybrids—A Biological Assessment

**DOI:** 10.3390/molecules27041315

**Published:** 2022-02-15

**Authors:** Paloma Begines, Sergio Martos, Irene Lagunes, Inés Maya, José M. Padrón, Óscar López, José G. Fernández-Bolaños

**Affiliations:** 1 Departamento de Química Orgánica, Facultad de Química, Universidad de Sevilla, Apartado 1203, E-41071 Seville, Spain; pbegines@us.es (P.B.); smardel251@iespinorueda.com (S.M.); imaya@us.es (I.M.); 2 BioLab, Instituto Universitario de Bio-Orgánica “Antonio González” (IUBO-AG), Universidad de La Laguna, c/Astrofísico Francisco Sánchez 2, E-38206 La Laguna, Spain; roslagunes@uv.mx

**Keywords:** organoselenium, isoselenoureas, selenocarbamates, isoselenocarbamates, polyphenols, antioxidant, GPx mimetic, antiproliferative agents

## Abstract

Being aware of the enormous biological potential of organoselenium and polyphenolic compounds, we have accomplished the preparation of novel hybrids, combining both pharmacophores in order to obtain new antioxidant and antiproliferative agents. Three different families have been accessed in a straightforward and chemoselective fashion: carbohydrate-containing *N*-acylisoselenoureas, *N*-arylisoselenocarbamates and *N*-arylselenocarbamates. The nature of the organoselenium framework, number and position of phenolic hydroxyl groups and substituents on the aromatic scaffolds afforded valuable structure–activity relationships for the biological assays accomplished: antioxidant properties (antiradical activity, DNA-protective effects, Glutathione peroxidase (GPx) mimicry) and antiproliferative activity. Regarding the antioxidant activity, selenocarbamates **24**–**27** behaved as excellent mimetics of GPx in the substoichiometric elimination of H_2_O_2_ as a Reactive Oxygen Species (ROS) model. Isoselenocarbamates and particularly their selenocarbamate isomers exhibited potent antiproliferative activity against non-small lung cell lines (A549, SW1573) in the low micromolar range, with similar potency to that shown by the chemotherapeutic agent cisplatin (*cis*-diaminodichloroplatin, CDDP) and occasionally with more potency than etoposide (VP-16).

## 1. Introduction

Organoselenium derivatives account for one of the most fascinating families of organic compounds. This is due not only to their high structural diversity [1], but also to the plethora of applications of such compounds. Since the isolation of selenoproteins, a subset of more than 20 proteins present in the three domains of life [2], and the consideration of selenium as an essential micronutrient [3], the interest in seleno-derivatives has incessantly increased [4].

Selenium-containing compounds are widely used both as key building blocks [5] in modern organic synthesis and as catalysts [6,7], providing in many cases chemo- [8], regio- [9] and stereoselective [10] pathways; even the precepts of Green Chemistry have been implemented in organoselenium chemistry [11].

The special non-covalent interactions that this kind of compound can exert [12], including strong H-bonding and inter/intramolecular chalcogen bonding interactions (in the family of σ-hole interactions) [13,14,15] give access to novel uses of organoselenium compounds, including as anion recognition [16], as chirality probes [17], or in crystal engineering [18], among others.

One of the most recognizable properties of selenium-containing compounds is their strong antioxidant character [19], as they are capable of scavenging reactive oxygen and nitrogen species (ROS and Reactive RNS, respectively). Prolonged exposure to high levels of such species (oxidative stress) [20] causes severe damage to organs and tissues as a result of their pro-oxidant character. This is a common hallmark [21] of numerous diseases and disorders such as diabetes, cancer, neurodegenerative, or cardiovascular diseases. Some organoselenium derivatives have been found to upregulate the endogenous antioxidant self-defence machinery for struggling pathological oxidative stress, and they have been proven to exert a cytoprotective action [22].

Selenium-containing compounds are endowed with a myriad of biological properties, such as antiviral [23], antibacterial [24], antiprotozoal [25], anti-inflammatory [26], or anti-Alzheimer’s [27] agents; potential therapeutic effects have been exhibited by seleno-derivatives acting as enzyme inhibitors [28]. Nonetheless, the most intensively studied property is undoubtedly their anti-cancer activity [29,30]. It has been reported that organoselenium compounds are usually featured with reduced toxicity when compared to inorganic counterparts [31]. Frequently reported motifs exhibiting antitumour properties are isoselenocyanates [32], selenocyanates [33,34,35], selenides/diselenides [36,37], selenoureas [33,38,39], or selenoheterocycles [40]. Selenium nanoparticles (SeNPs) are also gaining great attention as nano-sized drugs and drug delivery systems particularly effective against multidrug resistant tumours (MDR) [41]. Moreover, the use of organoselenium compounds has also been proven to be useful in adjuvant therapies [42], co-administered with a classical chemotherapeutic agent.

Herein, we report the chemoselective preparation of novel hybrid derivatives upon combination of organoselenium and polyphenolic scaffolds. Polyphenols are ubiquitous phytochemicals, widely distributed within the plant kingdom, where they are biosynthesized as secondary metabolites. Similar to selenium derivatives, polyphenols are endowed with relevant antioxidant and many other biological properties [43,44] and can be considered as privileged structures in medicinal chemistry. 

We will analyse the influence of the organoselenium framework (isoselenourea, selenocarbamate, isoselenocarbamate), the number of phenolic hydroxyl groups and the nature of the aromatic substituents on the antioxidant and antiproliferative properties of compounds prepared herein.

## 2. Results and Discussion

### 2.1. Chemistry

Stimulated by the exceptional properties of organoselenium and polyphenol compounds, we decided to explore new combinations of such pharmacophores with the aim of developing potential antioxidant and antiproliferative agents. The simultaneous presence of these two redox-active fragments could exhibit a synergic effect and modulate the bioactivities. The combination of two redox centres (quinone and arylselenide) has been recently reported in cytotoxic agents [37].

**Figure 1:**
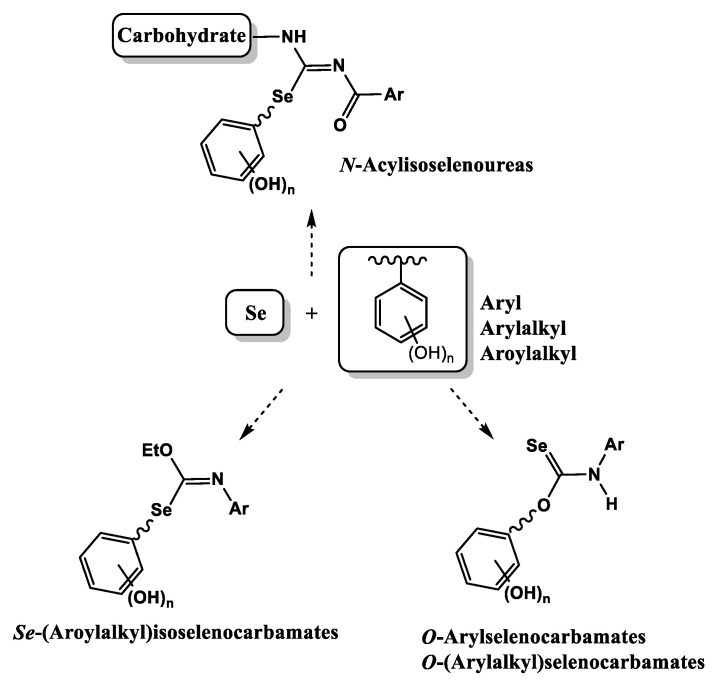
General structure of the organoselenium–polyphenol hybrids prepared herein.

Based on the strong antiproliferative features of selenoureas prepared in our group [38,39], we envisioned the possibility of transforming the selenoureido motif into isoselenoureas and analysing the influence of its structure on the biological properties (Figure 1). We have also incorporated carbohydrate residue; due to the Warburg effect, tumour cells overexpress sugar receptors (e.g., GLUT1); thus, appendages of glycosylated fragments might increase the chance of the antitumour drug of penetrating into the malignant cell [45]. Moreover, use of an aroyl scaffold has been claimed to be useful in the design of pro-drugs, as it is a cleavable motif under physiological conditions [46]. Additionally, polyphenolic selenocarbamates and *O*-alkyl isoselenocarbamates will also be attempted (Figure 1).

**Scheme 1:**
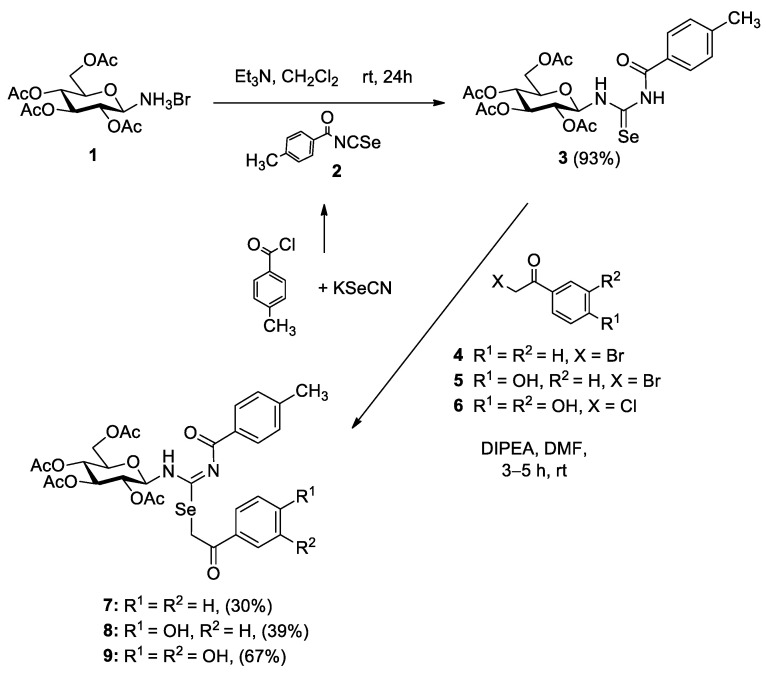
Preparation of isoselenoureas **7**–**9**.

We have carried out the preparation of the new phenolic isoselenoureas **7**–**9** (Scheme 1) using *N*-acyl selenourea **3** as the key synthetic intermediate. Treatment of per-*O*-acetyl β-d-glucopyranosylamine hydrobromide **1** [47] with *p*-methylbenzoyl isoselenocyanate **2**, in turn obtained by the reaction [48] of *p*-methylbenzoyl chloride with KSeCN, furnished **3** in an almost quantitative yield (93%). Spectroscopic data of **3** are in agreement with the proposed structure. Thus, the resonance at 11.66 ppm in 1H-NMR, assigned to the NH’ is clear evidence for an intramolecular hydrogen bonding involving the acyl group. Furthermore, the signal at 185.6 ppm in 13C-NMR is typical for the C = Se moiety in selenoureas [49].

Transformation into the aforementioned isoselenoureas **7**–**9** took place by *Se*-alkylation using phenacyl, 4-hydroxyphenacyl and 3,4-dihydroxyphenacyl halides **4**–**6** as the alkylating agents (Scheme 1) under mild basic conditions (diisopropylethylamine, DIPEA) following a S_N_2 process. ^13^C-NMR resonances ranging from 176.5 to 178.0 ppm in derivatives **7**–**9** also agree with reported data for sugar-derived *Se*-alkyl isoselenoureas [49]. The preference for the *Se*-alkylation over *N*-alkylation could be explained using the Pearson’s hard–soft acid–base (HSAB) theory [50]; alkyl halides, including phenacyl halides, are reported to be soft electrophilic species, in contrast with acyl halides, which are considered as hard electrophiles [51]. Therefore, it is the chalcogen atom (S, Se), the softer nucleophile, that becomes alkylated with alkyl halides in both thioxo [52] and selenoxo [53] derivatives.

In the outcome of the reaction, we also detected a series of phenacyl-derived selenides and diselenides as side-products resulting from the partial hydrolysis of compounds **7**–**9**, which could explain the moderate yields obtained in some cases after chromatographic purification (30–67%).

**Scheme 2:**
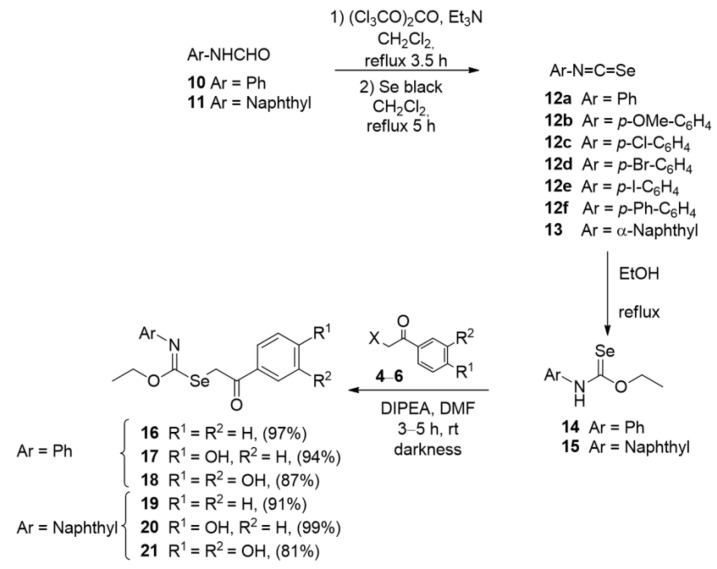
Preparation of iselenocarbamates **16**–**21**.

We also envisioned the possibility of preparing isoselenocarbamates **16**–**21** via a nucleophilic displacement involving selenocarbamates and phenacyl halides (Scheme 2); for that purpose, *N*-aryl-*O*-ethylselenocarbamates **14** and **15** [54] were selected as model compounds. To the best of our knowledge, no reported data on the *Se*-alkylation of such compounds by phenacyl halides can be found in literature. Asanuma et al. reported [55] the synthesis of *Se*-butyl isoselenocarbamates by treatment of an in situ generated isoselenocyanate with an alkoxide, followed by trapping of the corresponding transient oxyimidoylselenoate with BuI.

Selenocarbamates were in turn prepared by refluxing the corresponding parent isoselenocyanates **12** and **13** in EtOH. Heterocumulenes were prepared following a modification developed [49] in our group of the original procedure reported by Barton and co-workers [56]. Such improved methodology consists of the replacement of hazardous phosgene by easily handled triphosgene in the dehydration step of formamides (**10**, **11**) to give a transient non-isolated isocyanide. The latter intermediate reacts with black selenium to furnish the corresponding isoselenocyanates (Scheme 2).

The remarkable high yields obtained for the hitherto unknown isoselecarbamates **16**–**21** (81–99%) confirm the excellent nucleophilic properties of selenocarbamates towards α-haloketones. Regarding the spectral properties, a significant shielding for the C–Se resonance was found in all the isoselenocarbamates (152.7–155.5 ppm for **16**–**18**; 152.2–153.9 ppm for **19**–**21)** compared to parent selenocarbamates **14** and **15** (191.5 and 193.1 ppm, respectively) [54].

**Scheme 3:**
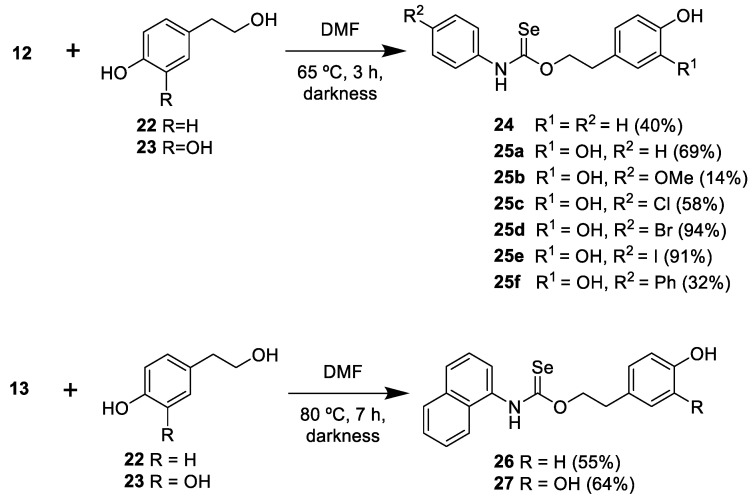
Preparation of *O*-tyrosyl and hydroxytyrosyl selenocarbamates **26**, **27**.

Finally, the one-step and chemoselective preparation of *O*-tyrosyl and *O*-hydroxytyrosyl selenocarbamates was also accomplished by direct nucleophilic coupling between the fully unprotected phenolic derivatives and heterocumulenes **12** and **13** (Scheme 3). Solventless conditions were first attempted, but only extensive decomposition was observed. On the contrary, the addition of aliquots of DMF to homogenize the crude reaction medium, and heating at 65–80 °C for 3–7 h furnished the phenolic selenocarbamates **24**–**27** from modest to excellent yields (14–94%). The reaction proceeded with total chemoselectivity, as only products coming from the nucleophilic addition of the aliphatic hydroxyl group were observed; therefore, no previous protection of the phenolic hydroxyl groups was required. Products underwent thermal degradation if subjected to higher temperatures, probably through a concerted mechanism leading to the release of an alkene and an aromatic isocyanide, as reported by Barton for simple selenocarbamates [56]. NMR spectra showed the presence of two rotamers due to the partial double character of the N–C bond on the selenocarbamato motif.

### 2.2. Biological Assays

The biological activities of *N*-acyl, *N*′-glycosylselenourea **3**, *Se*-phenacyl isoselenoureas **7**–**9**, *O*-ethyl isoselenocarbamates **16**–**21** and *N*-aryl *O*-phenetyl selenocarbamates **24**–**27** were assayed using different tests: antioxidant activity (DPPH assay, DNA oxidation inhibition and glutathione peroxidase-like activity), and antiproliferative activity against a panel of human cancer cell lines. The influence of the selenium-containing functional group, the number of phenolic hydroxyls and the nature of the *N*-aryl substituent on the exhibited biological properties has been analysed.

#### 2.2.1. Antioxidant Properties

##### DPPH Assay

The antiradical capacity of either naturally occurring or synthetic antioxidant agents is frequently evaluated using the DPPH (1,1-diphenyl-2-picrylhydrazyl) method [57], a stable and free radical used as a model. Such an assay provides a rapid screening of the in vitro radical scavenging properties of antioxidants that can quench DPPH by donating either a hydrogen atom or an electron. This provokes a decrease in the maximum absorbance of the solution (λ_max_ = 515 nm, deep purple colour) to furnish faded or yellowish solutions, depending on the concentration and potency of the antioxidant agent. Herein, a fixed concentration of DPPH (58.5 µM) was used, within the range of recommended values (25–70 µM), as highly concentrated DPPH solutions have been reported [58] to be far beyond the spectrophotometric accuracy, and thus should be avoided.

The antiradical potency of the tested compounds was quantified by calculating the EC_50_ (Table 1), that is, the concentration of the antioxidant required for reducing 50% of the initial DPPH concentration. Naturally occurring tyrosol (Tyr) **22** and hydroxytyrosol (HTyr) **23** together with BHT (butylated hydroxytoluene) as an example of a synthetic antioxidant have also been included in the assay for comparison.

Sugar-derived isoselenoureas **7**, **8** and isoselenocarbamates **16**, **17**, **19** and **20**, without phenolic hydroxyl groups, or bearing only one, showed negligible antiradical activity when tested at 250 µM concentrations (Table 1). The good antiradical activity depicted by sugar-derived selenourea **3**, lacking phenolic hydroxyl groups (EC_50_ = 22 ± 1 µM) can be attributed to the presence of a selenoureido moiety. This hypothesis is fulfilled considering isoselenoureas **7** and **8**, where the modified selenium-containing framework leads to a complete absence of activity; only when a catechol motif is present (derivative **9**), the good antiradical activity is restored (EC_50_ = 15 ± 1 µM). The positive influence of the catechol moiety is also demonstrated for *N*-aryl *O*-tyrosyl/hydroxytyrosyl selenocarbamates **24**–**27**; thus, a roughly five-fold increase in activity is found for catechol-containing derivatives **25** and **27** when compared with tyrosol-derived *O*-alkyl selenocarbamates **24** and **26**.

The best structural combination proved to be either an *N*-arylselenocarbamate or an isoselenocarbamate and a catechol motif (compounds **25c–f** (EC_50_ = 4.7–6.3 µM) and **18**/**21** (EC_50_ = 8.0, 7.8 µM, respectively). This represents up to a roughly three-fold and 15-fold increase in activity with respect to natural hydroxytyrosol **23** and synthetic BHT (Table 1).

##### DNA Oxidation Inhibition

Oxidative DNA damage, upon DNA exposition to free radicals, leads to the formation of a series of adducts, which in turn causes base mispairing, and thus mutations in the replication step [59]. Such deleterious effects have been correlated with the development of neurodegenerative diseases [60] such as Alzheimer’s and Parkinson’s and with carcinogenesis [61].

Potential DNA protection effects exerted by selenoureas, isoselenoureas, selenocarbamates and isoselenocarbamates prepared herein are depicted in Table 1. For this purpose, a modification of the methodology reported by Zhao and Liu [62] was used; in this procedure, the oxidative damage on DNA was simulated by homolytic decomposition of AAPH (2,2′-azobis(2-methylpropionamidine) dihydrochloride), a free radical generator, at physiological pH and temperature. Under these conditions, DNA degrades, leading to the formation of a series of carbonyl species which react with thiobarbituric acid to furnish the so-called thiobarbituric acid reactive species (TBARS), detectable spectrophotometrically. Tested compounds were assayed at a final concentration of 500 µM, and their activity was compared with that of tyrosol, hydroxytyrosol and BHT, well-known natural and synthetic antioxidant agents. The strongest inhibitors of DNA oxidative degradation were found to be the catechol-containing derivatives **9**, **18**, **21**, **25a** and **27**, with a similar potency (roughly 31–38% inhibition of DNA oxidation at the tested antioxidant concentration). 

Such results strongly suggest the requirement of the *o*-diphenol structure, and little influence of the selenium functionality; furthermore, the activity was found to be similar to that measured for hydroxytyrosol (40%) and remarkably higher than the ones obtained for tyrosol and BHT (18% and 4%, respectively), which behaved as poor inhibitors of the DNA degradation. Only selenocarbamate **26**, derived from tyrosol, had a relevant DNA-protecting activity among those derivatives lacking a catechol functionality.

##### Glutathione Peroxidase-like Activity

GPx refers to an ample family of enzymes which, among some other roles, act as efficient detoxifying agents catalysing the reduction of H_2_O_2_ and fatty acid hydroperoxides to water or alcohols, respectively. Of particular interest are GPx1-4, present in mammals, that have in common the presence of a selenocysteine fragment in the active site as the key redox component; GPx1-3 exert their activity in aqueous medium, whereas GPx4 protects lipidic membranes [63]. Therefore, the aforementioned GPxs are involved in balancing redox homeostasis and thus in the control of cellular oxidative stress.

Some selenium-containing derivatives have been reported to exhibit GPx-like activity [64,65,66]. We have evaluated the GPx mimicry activity exerted by the selenoderivatives prepared herein, following the procedure reported by Iwaoka and Kumakura [67]. Such a procedure involves the use of the organoselenium derivative in a sub-stoichiometric ratio, mimicking the activity exerted by the enzyme, and (±)-dithiothreitol (DTT) as the cofactor instead of gluthathione, used by the native enzyme. The formation of the corresponding cyclic disulphide, the oxidized form of DTT, was monitored by ^1^H-NMR spectroscopy (δ_DTTred_ = 3.7 ppm, δ_DTTox_ = 3.5 ppm), using CD_3_OD as a solvent; the capacity of the compounds to act as GPx mimetics is inversely proportional to t_1/2_, that is, the time required for reducing the initial DTT concentration to half of its value (Table 2). 

Compounds were initially tested in a 5% molar ratio, and a strong dependence of the nature of the organoselenium function and the number of hydroxyl groups was found on the GPx-like activity. Thus, selenourea **3** and isoselenoureas **7** and **8** behaved as good GPx mimetics, with t_1/2_ ranging from 3.4 to 5.9 min; for isoselenoureas **7**–**9**, a clear impairment of activity was observed with an increasing number of phenolic hydroxyl groups, leading to the complete absence of activity for **9** at the tested concentration. Conversely, for isoselenocarbamates **16**–**21**, the opposite behaviour was found; *N*-phenyl and *N*-naphthyl derivatives exhibited a similar potency, and in both cases, the maximum activity was reached with the catechol fragment (compounds **18** and **21**, t_1/2_ = 2.8 and 3.4 min, respectively).

The most potent GPx mimetics were found to be Tyr- and HTyr-derived selenocarbamates **24**–**27**; the extremely high rate of the reaction for *N*-phenyl-*O*-tyrosyl selenocarbamate **24** (t_1/2_ < 0.5 min) even precluded the measurement. Moreover, compounds **25a** and **27** also exhibited a low t_1/2_ value (1.0 and 0.9 min, respectively), as after 2 min of reaction, more than 85% conversion was reached. Accordingly, t_1/2_ values obtained at 1% molar concentration for **24**–**27** are also provided in Table 2 in order to compare their activities in a more precise fashion; such data suggest that *N*-phenyl counterparts **24** and **25** are the most potent derivatives, and a slight impairment in the activity takes place upon increasing the number of hydroxyl groups.

Remarkably, neither the background reaction (in the absence of organoselenium catalyst) nor the potent antioxidant hydroxytyrosol showed appreciable activity, demonstrating the importance of an organoselenium framework for introducing GPx-like activity in the phenolic derivatives.

These results reinforce the positive effects observed for the antioxidant properties of building up hybrid structures that simultaneously contain an organoselenium motif and a phenolic fragment, with improved properties compared to parent building blocks. Regarding DPPH, simple *O*-ethyl-*N*-arylselenocarbamates **14** and **15** exhibited only modest activity (EC_50_ = 148 ± 18 and 43 ± 7 µM, respectively) [54]; concerning simple phenolic compounds, the presence of an increasing number of phenolic hydroxyl groups was found to be positive for this activity. This is observed comparing Tyr **22** and BHT (EC_50_ > 500 and 70 ± 9 µM, respectively) with the catechol-containing HTyr **23** (EC_50_ = 15 ± 2 µM). An increasing number of hydroxyls in hybridized derivatives **16**–**18**, **19**–**21**, **26**, **27** clearly afforded increased activities, with improved properties to HTyr in most of the cases. 

A parallel effect was observed for the capacity of mimicking the activity of GPx for the scavenging of peroxides. Phenolic derivatives, lacking organoselenium functionalities in their structure (e.g., HTyr **23**), completely lacked such activity. Conversely, organoselenium compounds without phenolic hydroxyl groups exhibited, in most of the cases, good GPx mimicry activity (Table 2, e.g., **3**, **7**). For *O*-phenacyl isoselenocarbamates, the presence of one, or more particularly, two phenolic hydroxyl groups provoked a remarkable improvement in their activities (**16**–**18** and **19**–**21** series). Derivatives **16** and **19** shifted from t_1/2_ = 233 min, or no activity, respectively, to 2.8 and 3.4 min, respectively, for their catechol-containing counterparts in the capacity of scavenging peroxides.

#### 2.2.2. Antiproliferative Activity

**Figure 2:**
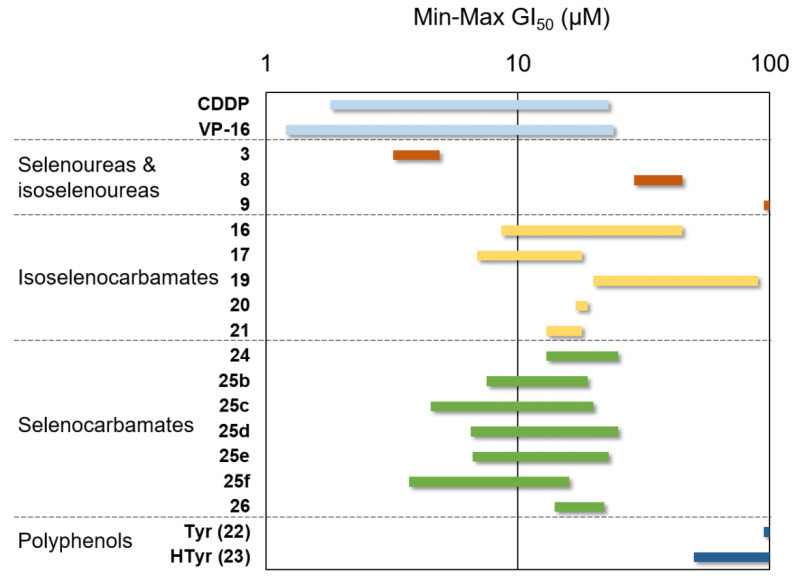
GI_50_ range plot of selenoureas, isoselenoureas, isoselenocarbamates and selenocarbamates against human solid tumour cell lines. Cyan: reference drugs; red: selenoureas & isoselenoureas; yellow: isoselenocarbamates; green: selenocarbamates; blue: polyphenols.

The four different families of selenoderivatives prepared herein (selenourea **3**, isoselenoureas **8**, **9**, isoselenocarbamates **16**, **17**, **19**–**21**, and selenocarbamates **24**–**26**) were tested in vitro against a panel of six human solid tumour cell lines: A549 (non-small cell lung), HBL-100 (breast), HeLa (cervix), SW1573 (non-small cell lung), T-47D (breast) and WiDr (colon), the latter two as examples of drug-resistant lines. The precursor tyrosol **22** and hydroxytyrosol **23**, together with the standard anticancer drugs etoposide (VP-16) and cisplatin (CDDP) as reference compounds, were also included in the study for comparison. The NCI protocol was used with minor modifications [68], and data, expressed as GI_50_ values (µM), are depicted in Table 3 and Figure 2.

Compounds could be categorized into four different groups according to their antiproliferative activity: strong (GI_50_ < 10 µM), good (GI_50_ 10–20 µM), moderate (GI_50_ 20–100 µM), and inactive (GI_50_ > 100 µM). Overall, most compounds prepared herein were active, exhibiting growth inhibition in all tumour cell lines, unlike isoselenourea **9** and precursors Tyr (**22**) and HTyr (**23**), which turned out to be inactive, or almost inactive.

Interesting structure–activity relationships can be established upon data analysis: *N*-acylselenourea 3 turned out to be the strongest antiproliferative agent based on the GI_50_ range (lower than 5.0 µM in all cell lines); this compound exhibited a comparable potency to CDDP in A549, HBL-100 and Hela cell lines, and improved activity (roughly 5.0-fold) for the multidrug-resistant lines. 

Conversion of **3** into Se-phenacyl isoselenoureas clearly provoked an impairment of the activity; thus a 10-fold decrease in activity was found for **8**, and complete abolishment of activity for **9** (Table 3). These results show a pivotal influence of the nature of the organoselenium motif on antiproliferative activity, and they suggest that the selone functionality (C=Se) could be crucial.

Isoselenocarbamates **16**–**21** were also accessed, where the influence of both the nature of the *N*-aryl system and the number of phenolic hydroxyl groups could be analysed. Incorporation of one hydroxyl group (**17** vs. **16**; **20** vs. **19**) had a clear positive effect on activity; however, the presence of one or two hydroxyl groups (catechol moiety) did not seem to have much influence, as the activity of derivatives **20** (GI_50_ 15–21 µM) and **21** (GI_50_ 13–18 µM) was found to be virtually the same.

Additionally, the use of an *N*-phenyl substituent compared to N-naphthyl also improved activity, particularly against A549, HBL-100 and T-47D lines.

Regarding selenocarbamates **24**–**27**, the optimal pharmacophore turned out to be a catechol motif, and an N-phenyl scaffold bearing substituents with electron-withdrawing effects, such as halogens or a biphenyl motif (**25c**–**f**), particularly the former (Table 3). Halogen-containing selenocarbamates **25c**–**f** exhibited a certain selectivity towards lung cancer cell lines (A549 and SW1573), behaving as strong antiproliferative agents (GI_50_ 4.5–9.0 and 5.9–6.6 µM, respectively). Roughly, 2–4-fold enhanced activity was achieved for those compounds in SW1573 in comparison with VP-16.

Once again, the benefits from the preparation of hybridized structures can also be observed in the measured antiproliferative activities (Table 3). Thus, whereas phenolic derivatives (Tyr, HTyr) were found to be either inactive or with modest activities against tumour cell lines, an increasing number of phenolic hydroxyl groups of the hybridized derivatives exceeded the activity of the non-phenolic counterparts (e.g., **16** vs. **17**, **19** vs. **20**, **21**).

## 3. Materials and Methods

### 3.1. General Procedures

Optical rotations were measured with a Jasco P-2000 polarimeter (Cambridge, MA, USA). ^1^H (300.1 and 500.1 MHz) and ^13^C (75.5 and 125.7 MHz) NMR spectra were recorded on Bruker Avance-300 and Avance-500 spectrometers (Bruker Biospin, Rheinstetten, Germany) at rt. The assignments of ^1^H and ^13^C signals were confirmed by homonuclear COSY (Correlated Spectroscopy) and heteronuclear 2D correlated spectra, respectively. ^1^H and ^13^C plots are included in the Supplementary Materials.

Mass spectra, CI (Chemical Ionization) and LSI (Liquid Secondary Ionization) were recorded on AutoSpec-Q mass spectrometer (Micromass UK Ltd., Manchester, UK) with a resolution of 1000 or 10,000 (10% valley definition). For LSI spectra, ions were produced by a beam of xenon atoms and Cs^+^ ions, respectively, using thioglycerol or *o*-nitrobenzyl alcohol as matrices and NaI as additive. TLC (Thin Layer Chromatography) was performed on aluminium pre-coated sheets (E. Merck Silica gel 60 F_254_); spots were visualized by UV (Ultraviolet) light, by charring with 3% ninhydrin in EtOH, or with 10% vanillin in EtOH containing 1% of H_2_SO_4_. Column chromatography was performed using E. Merck silica gel 60 (40–63 µm).

The antioxidant assays were performed in a Hitachi U-2900 spectrophotometer (Hitachi Europe Ltd., Stoke Poges, UK), using PS (Polystyrene) cuvettes (1 × 1 × 4.5 cm) and the blank indicated in each case.

### 3.2. Antioxidant Assays

#### 3.2.1. Free Radical-Induced DNA Degradation Inhibition

The capacity of the organoselenium derivatives to ameliorate the free radical-induced oxidation of DNA was analysed using AAPH as the free radical initiator, following a modification of the procedure reported by Zhao and Liu [62]. A methanolic solution of the tested compound (10.0 mM, 100 µL), or pure MeOH for the control, and 0.1 M phosphate buffer (1.9 mL, pH 7) containing AAPH (40 mM final concentration) and DNA (2.0 mg/mL final concentration) in screw-cap Pyrex^®^ test tubes was incubated at 37 °C for 5 h. After that, the reaction was stopped by cooling it down to 0 °C; to the cooled solutions, 1.0 mL of a TBA (thiobarbituric acid) solution (1.0 g of TBA and 0.4 g of NaOH in 100 mL of phosphate buffer solution) and 3% aq. TCA (trichloroacetic acid, 1.0 mL) were added. The tubes were heated at 100 °C for 15 min; after cooling down again, *n*-butanol (2.0 mL) was added, and they were vigorously stirred for 10 s in a vortex. The mixture was decanted for 15–30 min, the organic phase was separated, and the absorbance was measured at 535 nm against an *n*-butanol blank. Results are expressed as the percentage of inhibition of the DNA degradation according to the expression:

(1) $$\%\mathrm{Inhibition}=\frac{A_{\mathrm{control}}-A_{\mathrm{sample}}}{A_{\mathrm{control}}}\times 100$$

A_control_ and A_sample_ refer to the absorbance of the control and sample solutions, respectively. Tyr, HTyr and BHT were included as references.

#### 3.2.2. Glutathione Peroxidase Mimicry

The capacity of the organoselenium derivatives to act as glutathione peroxidase mimetics was measured with ^1^H-NMR spectroscopy, following the procedure reported by Iwaoka and Kumakura [67]. To a solution of DDT^red^ (25 mg, 0.161 mmol) and the organoselenium derivative (0.05 or 0.01 molar equiv.) in CD_3_OD (0.6 mL), 30% H_2_O_2_ (18 µL, 0.176 mmol) was added. The disappearance of DTT^red^ C*H*OH protons (3.68 ppm) and the appearance of DTT^ox^ C*H*OH protons (3.51 ppm) were monitored at different time intervals. The DTT^red^/ DTT^ox^ ratio was obtained by integration of the above-mentioned signals in order to calculate t_1/2_, that is, the time required to reduce DTT^red^ to 50% of its initial concentration. A control experiment, in the absence of the organoselenium derivative, was also carried out in order to evaluate the background reaction velocity, which turned out to be negligible.

#### 3.2.3. Statistical Analysis

All tests were run in triplicate. Values are expressed as the confidence interval, which was calculated for *p* = 0.95 using the Student’s t distribution. 

### 3.3. Chemistry

*N*-(4-Methylbenzoyl)-*N*^′^-(2,3,4,6-tetra-*O*-acetyl-β-d-glucopyranosyl)selenourea (**3**). To a solution of *p*-methylbenzoyl isoselenocyanate **2** (361 mg, 1.61 mmol, 1.5 equiv.) in dry CH_2_Cl_2_ (20 mL) were added 2,3,4,6-tetra-*O*-acetyl-β-d-glucopyranosylamine hydrobromide **1** (465 mg, 1.09 mmol) and Et_3_N (152 μL, 1.09 mmol, 1.0 equiv.). Reaction was kept stirring at rt, under Ar in the dark for 24 h. Then, it was concentrated to dryness, and the residue was purified by column chromatography (hexane → 2:1 hexane-EtOAc) to give **3** as a yellow solid (575 mg, 92%); mp: 165–167 °C (EtOH); *R*_F_ 0.22 (1:2 EtOAc-hexane); ${\left[\alpha \right]}_{D}^{24}$ + 16 (*c* 1.07, CH_2_Cl_2_); ^1^H-NMR (300 MHz, CDCl_3_) δ 11.66 (d, 1H, *J*_NH’,H1_ = 9.0 Hz, NH’), 9.43 (s, 1H, NH), 7.75 (m, 2H, Ar-H*o*), 7.31 (m, 2H, Ar-H*m*), 5.84 (brt, 1H, *J*_1′,2′_ = 9.0 Hz, H-1′), 5.39 (t, 1H, *J*_2′,3′_= *J*_3′,4′_ = 9.3 Hz, H-3′), 5.24 (t, 1H, H-2′), 5.13 (dd, 1H, *J*_4′,5′_ = 10.1 Hz, H-4′), 4.30 (dd, 1H, *J*_5′,6′a_ = 4.6 Hz, *J*_6′a,6′b_ = 12.5 Hz, H-6′a), 4.16 (dd, 1H, *J*_5′,6′b_ = 2.1 Hz, H-6′b), 3.90 (ddd, 1H, H-5′), 2.43 (s, 3H, PhC*H*_3_), 2.10, 2.07, 2.04, 2.03 (4s, 3H each, 4OAc) ppm; ^13^C-NMR (75.5 MHz, CD_3_OD) δ 185.6 (CSe), 170.8, 170.2, 170.1, 169.6 (4OAc), 166.3 (Ar*C* = O), 145.3 (Ar-C*p*), 130.0 (Ar-C*o*), 128.1 (Ar-C*ipso*), 128.0 (Ar-C*m*), 85.6 (C-1), 74.1 (C-5), 72.9 (C-3), 70.4 (C-2), 68.3 (C-4), 61.7 (C-6), 21.8 (*C*H_3_Ar), 20.9–20.7 (4*C*H_3_CO) ppm; HRCI-MS *m/z* calcd. for C_23_H_28_N_2_O_10_^80^Se ([M]^+^): 572.0904, found: 572.0901.

#### 3.3.1. General Procedure for the Synthesis of Se-Phenacyl Isoselenoureas **7**–**9**

To a solution of selenourea **3** (100 mg, 0.18 mmol) in dry DMF (5 mL) were added the corresponding phenacyl halide (0.21 mmol, 1.2 equiv.) and DIPEA (45 μL, 0.26 mmol, 1.40 equiv.). The corresponding mixture was stirred at rt, under Ar in the dark for 3–5 h. Then, it was concentrated to dryness and the residue was purified by column chromatography (hexane → 2:1 hexane-EtOAc) to give *Se*-phenacyl isoselenoureas **7**–**9** as amorphous solids.

*N*-(*p*-Methylbenzoyl)-*Se*-phenacyl-*N′*-(2,3,4,6-tetra-*O*-acetyl-β-d-glucopyranosyl)isoselenourea (**7**). Phenacyl bromide **4** (41 mg, 0.21 mmol) was used. Compound **7** was obtained as a syrup. Yield: 36 mg, 30%; *R*_F_ 0.28 (1:2 EtOAc-hexane); ${\left[\alpha \right]}_{D}^{24}$ −51 (*c* 1.00, CH_2_Cl_2_); ^1^H-NMR (300 MHz, CDCl_3_) δ 8.89 (brs, 1H, NH), 8.07 (m, 2H, Ar-H), 7.97 (m, 2H, Ar-H), 7.63 (m, 1H, Ar-H), 7.50 (m, 2H, Ar-H), 7.23 (m, 2H, Ar-H), 5.78 (brs, 1H, H-1′), 5.42 (t, 1H, *J*_2′,3′_ = *J*_3′,4′_ = 9.3 Hz, H-3′), 5.32 (t, 1H, *J*_1′,2′_ = 9.3 Hz, H-2′), 5.19 (t, 1H, *J*_4′,5′_ = 10.0 Hz, H-4′), 4.27 (dd, 1H, *J*_5′,6′a_ = 5.5 Hz, *J*_6′a,6′b_ = 12.6 Hz, H-6′a), 4.18 (dd, 1H, *J*_5′,6′b_ = 2.2 Hz, H-6′b), 4.06 (brs, 2H, CH_2_Se), 3.90 (ddd, 1H, H-5′), 2.41 (s, 3H, PhC*H*_3_), 2.07, 2.06, 2.05, 1.96 (4s, 3H each, 4OAc) ppm; ^13^C-NMR (75.5 MHz, CDCl_3_) δ 176.5 (N = *C*–Se), 170.9, 170.4, 170.2, 169.6 (4*C*O), 161.0 ( = NCO-) 143.4 (Ar-C*p* benzoyl), 134.8 (Ar-C*ipso* benzoyl), 134.4 (Ar-C*p* phenacyl), 133.1 (Ar-C*ipso* phenacyl), 130.1 (Ar-C*o* benzoyl), 129.1 (Ar-C*o* phenacyl) 129.1 (Ar-C*m* phenacyl), 129.0 (Ar-C*m* benzoyl), 77.4 (C-1′), 74.1 (C-5′), 73.5 (C-3′), 71.0 (C-2′), 68.7 (C-4′), 62.2 (C-6′), 28.6 (CH_2_Se), 21.9 (*C*H_3_Ar), 20.9–20.7 (4*C*H_3_CO) ppm; HRLSI-MS *m/z* calcd. for C_31_H_35_N_2_O_11_^80^Se ([M + H]^+^): 691.1401, found: 691.1387.

*Se*-(4-Hydroxyphenacyl)-*N*-(4-methylbenzoyl)-*N′*-(2,3,4,6-tetra-*O*-acetyl-β-d-glucopyranosyl)isoselenourea (**8**). 4-Hydroxyphenacyl bromide **5** (45 mg, 0.21 mmol) was used. Yield: 48 mg, 39%; mp: 138–140 °C (MeOH); *R*_F_ 0.40 (1:1 EtOAc-hexane); ${\left[\alpha \right]}_{D}^{24}$ +6 (*c* 1.00, CH_2_Cl_2_); ^1^H-NMR (500 MHz, CDCl_3_) δ 10.44 (brd, 1H, *J*_NH,H1′_ = 7.0 Hz, NH’), 8.05 (m, 2H, Ar-H), 7.78 (m, 2H, Ar-H), 7.31 (m, 2H, Ar-H), 7.08 (m, 2H, Ar-H), 5.36 (t, 1H, *J*_2′,3′_ = *J*_3′,4′_ = 9.5 Hz, H-3′), 5.31 (t, 1H, *J*_1′,2′_ = 8.9 Hz, H-1′), 5.19 (t, 1H, H-2′), 5.12 (t, 1H, *J*_4′,5′_ = 10.1 Hz, H-4′), 4.24 (dd, 1H, *J*_5′,6′a_ = 4.7 Hz, *J*_6′a,6′b_ = 12.4 Hz, H-6′a), 4.17 (dd, 1H, *J*_5′,6′b_ = 2.1 Hz, H-6b’), 3.86 (ddd, 1H, H-5′), 2.65 (s, 2H, CH_2_Se), 2.32 (s, 3H, PhC*H*_3_), 2.06, 2.04, 2.04, 2.03 (4s, 3H each, 4OAc) ppm; ^13^C-NMR (125.7 MHz, CDCl_3_) δ 197.0 (Ar*C*OCH_2_), 178.0 (N = *C*–Se), 170.7, 170.2, 169.8, 169.5 (4OAc), 161.0 (=N–CO), 155.3 (Ar-C*p* phenacyl), 143.3 (Ar-C*p* benzoyl), 135.0, 133.7 (ArC*ipso*, benzoyl, phenacyl), 129.9 (Ar-C*o* phenacyl), 129.8 (ArC-*o* benzoyl), 128.9 (Ar-C*m* benzoyl), 122.4 (Ar-C*m* phenacyl), 80.5 (C-1′), 73.9 (C-5′), 72.9 (C-3′), 70.6 (C-2′), 68.3 (C-4′), 62.0 (C-6′), 26.8 (CH_2_Se), 21.7 (*C*H_3_Ar), 20.8–20.6 (4*C*H_3_CO) ppm; HRLSI-MS *m/z* calcd. for C_31_H_34_N_2_NaO_12_^80^Se ([M + Na]^+^): 729.1169, found: 729.1171.

*Se*-(3,4-Dihydroxyphenacyl)-*N*-(4-methylbenzoyl)-*N′*-(2,3,4,6-tetra-*O*-acetyl-β-d-glucopyranosyl)isoselenourea (**9**). 3,4-Dihydroxyphenacyl chloride **6** (39 mg, 0.21 mmol) was used. Yield: 47 mg, 37%; mp: 79–81 °C (MeOH); *R*_F_ 0.20 (1:1 EtOAc-hexane); ${\left[\alpha \right]}_{D}^{24}$ + 9 (*c* 1.00, CH_2_Cl_2_); ^1^H-NMR (500 MHz, CDCl_3_) δ 10.66 (brs, 1H, NH), 7.87–7.82 (m, 4H, Ar-C*o* benzoyl, phenacyl), 7.20–7.12 (m, 3H, Ar-C*m* benzoyl, H-5 phenacyl), 5.39 (t, 1H, *J*_2′,3′_= *J*_3′,4′_ = 9.3 Hz, H-3′), 5.30–5.23 (m, 2H, H-1′, H-2′), 5.11 (t, 1H, *J*_4′,5′_ = 9.5 Hz, H-4′), 4.28 (dd, 1H, *J*_5′,6′a_ = 4.7 Hz, *J*_6′a,b_ = 12.6 Hz, H-6′a), 4.22 (dd, 1H, *J*_5′,6′b_ = 2.1 Hz, H-6′b), 3.86 (ddd, 1H, H-5′), 2.58 (s, 2H, CH_2_Se), 2.36 (s, 3H, PhC*H*_3_), 2.10, 2.09, 2.05, 2.05 (4s, 3H each, 4OAc) ppm; ^13^C-NMR (125.7 MHz, CDCl_3_) δ 196.0 (Ar*C*OCH_2_), 177.5 (N = *C*–Se), 170.8, 170.2, 170.1, 169.5 (4OAc), 161.0 ( = N–*C*O), 152.6 (C-4 phenacyl), 143.9 (Ar-C*p* benzoyl), 140.5 (C-3 phenacyl), 133.0, 130.7 (Ar-C*ipso* benzoyl, phenacyl), 129.8 (Ar-C*o* benzoyl), 129.2 (Ar-C*m* benzoyl), 128.9 (C-6 phenacyl), 123.2 (C-2 phenacyl), 119.2 (C-5 phenacyl), 80.9 (C-1′), 74.2 (C-5′), 72.7 (C-3′), 70.6 (C-2′), 68.2 (C-4′), 61.8 (C-6′), 26.5 (CH_2_Se), 21.7 (*C*H_3_Ar), 20.8–20.6 (4*C*H_3_CO) ppm.

#### 3.3.2. General Procedure for the Preparation of Se-Phenacyl Isoselenocarbamates **16**–**21**

To a solution of *O*-ethyl-*N*-phenylselenocarbamate **14** (110 mg, 0.48 mmol) or *O*-ethyl-*N*-(1-naphthyl)selenocarbamate **15** (134 mg, 0.48 mmol) in DMF (5 mL) was added the corresponding phenacyl halide **4**–**6** (1.0 equiv.) and DIPEA (84 µL, 0.48 mmol, 1.0 equiv.) The resulting mixture was stirred at rt and in the dark for 5 h. Then, it was concentrated to dryness and the residue was purified by column chromatography (hexane → 2:1 hexane-EtOAc) to give *Se*-phenacyl isoselenocarbamates **16**–**21**.

*O*-Ehyl-*Se*-phenacyl-*N*-phenylisoselenocarbamate (**16**). Phenacyl bromide **4** (96 mg, 0.48 mmol) was used. Compound **16** was obtained as an amorphous orange solid. Yield: 161 mg, 97%; *R*_F_ 0.24 (1:10 EtOAc-hexane); ^1^H-NMR (500 MHz, CDCl_3_) δ 7.96 (m, 2H, Ar-H*o*, phenacyl), 7.58 (m, 1H, Ar-H*p*, phenacyl), 7.47 (m, 2H, Ar-H*m*, phenacyl), 7.28 (m, 2H, Ar-H*m*, NPh), 7.09 (m, 1H, Ar-H*p*, NPh), 6.88 (m, 2H, Ar-H*o*, NPh), 4.42 (q, 2H, *J*_H,H_ = 7.1 Hz, CH_2_O), 4.34 (brs, 2H, CH_2_Se), 1.33 (t, 3H, CH_3_) ppm; ^13^C-NMR (125.5 MHz, CDCl_3_) δ 195.0 (CO), 152.7 (N = *C*–Se), 147.6 (Ar-C*_ipso_*, NPh), 135.9 (Ar-C*_ipso_*, phenacyl), 133.6 (Ar-C*p*, phenacyl), 129.2 (Ar-C*m*, NPh), 128.8 (Ar-C*m*, phenacyl), 128.7 (Ar-C*o*, phenacyl), 124.4 (Ar-C*p*, NPh), 121.6 (Ar-C*o*, NPh), 66.2 (CH_2_O), 30.9 (CH_2_Se), 14.2 (CH_3_) ppm; HRCI-MS *m/z* calcd. for C_17_H_18_NO_2_^80^Se ([M + H]^+^): 348.0497, found: 348.0499.

*O*-Ethyl-*Se*-(*p*-hydroxyphenacyl)-*N*-phenylisoselenocarbamate (**17**). 4-Hydroxyphenacyl bromide **5** (103 mg, 0.48 mmol) was used. Compound **17** was obtained as a white solid. Yield: 163 mg, 94%; mp: 95–97 °C (MeOH); *R*_F_ 0.36 (1:2 EtOAc-hexane); ^1^H-NMR (300 MHz, CD_3_OD) δ 7.78 (m, 2H, Ar-H*o*, ArOH), 7.26 (m, 2H, Ar-H*m*, NPh), 7.07 (m, 1H, Ar-H*p* NPh), 6.85 (m, 2H, Ar-H*m*, ArOH), 6.83 (m, 2H, Ar-H*o*, NPh), 4.35 (q, 2H, *J*_H,H_ = 7.1 Hz, CH_2_O), 4.34 (brs, 2H, CH_2_Se), 1.27 (s, 3H, CH_3_) ppm; ^13^C-NMR (75.5 MHz, CD_3_OD) δ 195.8 (CO), 164.2 (Ar-C*p*, ArOH), 155.4 (N = *C*–Se), 148.9 (Ar-C*_ipso_*, NPh), 132.4 (Ar-C*o*, ArOH), 130.1 (Ar-C*m*, NPh), 128.7 (Ar-C*_ipso_*, ArOH), 125.3 (Ar-C*p*, NPh), 122.6 (Ar-C*o*, NPh), 116.3 (Ar-C*m*, ArOH), 67.0 (CH_2_O), 31.4 (CH_2_Se), 14.3 (CH_3_) ppm; HRCI-MS *m/z* calcd for C_17_H_18_NO_3_^80^Se ([M + H]^+^): 346.0446, found: 346.0446.

*Se*-(3,4-Dihydroxyphenacyl)-*O*-ethyl-*N*-phenylisoselenocarbamate (**18**). 3,4-dihydroxyphenacyl chloride **6** (90 mg, 0.48 mmol) was used. Compound **18** was obtained as a syrup. Yield: 158 mg, 87%; *R*_F_ 0.26 (20:1 CH_2_Cl_2_–MeOH); ^1^H-NMR (500 MHz, CD_3_OD) δ 7.44 (dd, 1H, *J*_5,6_ = 8.1 Hz, *J*_2,6_ = 2.2 Hz, H-6, phenacyl), 7.42 (d, 1H, H-2, phenacyl), 7.27 (m, 2H, Ar-H*m*, NPh), 7.08 (m, 1H, Ar-H*p*, NPh), 6.85–6.82 (m, 3H, H-5 phenacyl, Ar-H*o*, NPh), 4.36 (q, 2H, *J*_H,H_ = 7.1 Hz, CH_2_O), 4.32 (brs, 2H, CH_2_Se), 1.28 (t, 3H, CH_3_) ppm; ^13^C-NMR (125.7 MHz, CD_3_OD) δ 196.0 (*C*O), 155.5 (N = *C*–Se), 152.6 (C-4, phenacyl), 149.0 (Ar-C*_ipso_*, NPh), 146.6 (C-3, phenacyl), 130.1 (Ar-C*m*, NPh), 129.3 (C-1, phenacyl), 125.3 (Ar-C*p*, NPh), 123.7 (C-6, phenacyl), 122.6 (Ar-C*o*, NPh), 116.4 (C-2, phenacyl), 115.8 (C-5, phenacyl), 67.0 (CH_2_O), 31.6 (CH_2_Se), 14.4 (CH_3_) ppm; HRCI-MS *m/z* calcd for C_17_H_18_NNaO_4_^80^Se ([M + H]^+^): 380.0396, found: 380.0398.

*O*-Ethyl-*N*-(α-naphthyl)-*Se*-phenacylisoselenocarbamate (**19**). Phenacyl bromide **4** (96 mg, 0.48 mmol) was used. Compound **19** was obtained as a yellow syrup. Yield: 173 mg, 91%; *R*_F_ 0.28 (1:10 EtOAc-hexane); ^1^H-NMR (300 MHz, CDCl_3_) δ 7.93 (m, 3H, H-5 or H-8, Naphthyl, Ar-H*o*, Ph), 7.82 (dd, 1H, *J*_H,H_ = 7.1 Hz, *J*_H,H_ = 1.9 Hz, H-5 or H-8, naphthyl), 7.61 (brd, 1H, *J*_3,4_ = 8.2 Hz, H-4, Naphthy), 7.57 (m, 1H, Ar-H*p*, Ph), 7.50–7.40 (m, 4H, H-6, H-7, Naphthyl, Ar-H*m*, Ph), 7.36 (dd, 1H, *J*_2,3_ = 7.3 Hz, *J*_3,4_ = 7.8 Hz, H-3, naphthyl), 6.98 (dd, 1H, *J*_2,4_ = 0.9 Hz, H-2, naphthyl), 4.62 (q, 2H, *J*_H,H_ = 7.1 Hz, CH_2_O), 4.34 (brs, 2H, CH_2_Se), 1.43 (t, 3H, CH_3_) ppm; ^13^C-NMR (75.5 MHz, CDCl_3_) δ 195.0 (CO), 153.2 (N = C–Se), 143.8 (C-1, naphthyl), 135.8 (C*_ipso_*_,_ Ph), 134.4 (C-4a, naphthyl), 133.6 (Ar-C*p*, Ph), 128.8 (Ar-C*m*, Ph), 128.7 (Ar-C*o*, Ph), 128.0, 127.4, 126.3, 125.8, 125.7, 124.6, 123.5 (C-3–C-8, C8a, naphthyl), 116.3 (C-2, Naphthyl), 66.4 (CH_2_O), 30.9 (CH_2_Se), 14.4 (CH_3_) ppm; HRCI-MS *m/z* calcd for C_21_H_19_NO_2_^80^Se ([M]^+^): 397.0576, found: 397.0583.

*O*-Ethyl-*Se*-(*p*-hydroxyphenacyl)-*N*-(α-naphthyl)isoselenocarbamate (**20**). 4-Hydroxyphenacyl bromide **5** (103 mg, 0.48 mmol) was used. Compound **20** was obtained as a white foam. Yield: 198 mg, quant.; *R*_F_ 0.27 (1:2 EtOAc-hexane); ^1^H-NMR (500 MHz, CDCl_3_) δ 7.92 (brd, 1H, *J*_H,H_ = 8.1 Hz, H-5 or H-8, naphthyl), 7.83 (m, 2H, Ar-H*o*, phenacyl), 7.80 (brd, 1H, *J*_H,H_ = 8.3 Hz, H-5 or H-8, naphthyl), 7.60 (brd, 1H, *J*_3,4_ = 8.2 Hz, H-4, naphthyl), 7.46, 7.42 (2td, 1H each, *J*_H,H_ = 8.2 Hz, *J*_H,H_ = 1.4 Hz, H-6, H-7, naphthyl), 7.35 (dd, 1H, *J*_2,3_ = 7.3 Hz, *J*_3,4_ = 7.8 Hz, H-3, naphthyl), 6.97 (dd, 1H, *J*_2,4_ = 1.0 Hz, H-2, naphthyl), 6.83 (m, 2H, Ar-H*m*, phenacyl), 6.53 (brs, 1H, OH), 4.61 (q, 2H, *J*_H,H_ = 7.1 Hz, CH_2_O), 4.28 (brs, 2H, CH_2_Se), 1.42 (t, 3H, CH_3_) ppm; ^13^C-NMR (125.7 MHz, CDCl_3_) δ 194.2 (CO), 161.0 (Ar-C*p*, phenacyl), 153.7 (N = C–Se), 143.7 (C-1, naphthyl), 134.5 (C-4a, naphthyl), 131.5 (Ar-C*o*, phenacyl), 128.6, 128.0, 127.4, 126.4, 125.8, 125.7, 124.7, 123.5 (C*_ipso_*, phenacyl, C-3–C-8, C8a, naphthyl), 116.5 (C-2, naphthyl), 115.7 (Ar-C*m*, phenacyl), 66.5 (CH_2_O), 30.7 (CH_2_Se), 14.4 (CH_3_) ppm; HRCI-MS *m/z* calcd for C_21_H_19_NO_3_^80^Se ([M]^+^): 413.0525, found: 413.0521.

*Se*-(3,4-Dihydroxyphenacyl)-*O*-ethyl-*N*-(α-naphthyl)isoselenocarbamate (**21**). 3,4-dihydroxyphenacyl chloride **6** (90 mg, 0.48 mmol) was used. Compound **21** was obtained as a yellow syrup. Yield: 167 mg, 81%; *R*_F_ 0.22 (20:1 CH_2_Cl_2_–MeOH); ^1^H-NMR (300 MHz, CDCl_3_) δ 7.92 (dd, 1H, *J*_H,H_ = 7.7 Hz, *J*_H,H_ = 1.5 Hz, H-5 or H-8, naphthyl), 7.80 (dd, 1H, *J*_H,H_ = 7.5 Hz, *J*_H,H_ = 1.6 Hz, H-5 or H-8, naphthyl), 7.59 (m, 2H, H-4 naphthyl, H-6 phenacyl), 7.49–7.38 (m, 3H, H-2 phenacyl, H-6, H-7, naphthyl), 7.35 (dd, 1H, *J*_2,3_ = 7.3 Hz, *J*_3,4_ = 7.7 Hz, H-3, Naphthyl), 7.16 (brs, 1H, OH), 6.97 (dd, 1H, *J*_2,4_ = 1.0 Hz, H-2, Naphthyl), 6.86 (d, 1H, *J*_5,6_ = 8.3 Hz, H-5, phenacyl), 6.70 (brs, 1H, OH), 4.58 (q, 2H, *J*_H,H_ = 7.1 Hz, CH_2_O), 4.23 (brs, 2H, CH_2_Se), 1.38 (t, 3H, CH_3_) ppm; ^13^C-NMR (75.5 MHz, CDCl_3_) δ 195.1 (CO), 153.9 (N = *C*–Se), 150.3 (C-4, phenacyl), 144.0 (C-3 phenacyl, C-1 naphthyl), 134.4, (C-4a, naphthyl), 128.5, 128.1, 127.4, 126.4, 125.8, 124.8, 123.7, 123.4 (C-1 and C-6, phenacyl; C-3–C-8, C8a, naphthyl), 116.6 (C-2, naphthyl), 115.5, 114.8 (C-2, C-5, phenacyl), 66.6 (CH_2_O), 30.6 (CH_2_Se), 14.4 (CH_3_) ppm; HRCI-MS *m/z* calcd for C_21_H_19_NO_4_^80^Se ([M]^+^): 429.0474, found: 429.0479.

#### 3.3.3. General Procedure for the Preparation of Selenocarbamates **24**, **25**

A mixture of tyrosol (26 mg, 0.19 mmol, 1.0 equiv.) or hydroxytyrosol (29 mg, 0.19 mmol, 1.0 equiv.) and the corresponding aryl isoselenocyanate (1.5 equiv. for **24**, **25a**; 1.25 equiv. for **25b**–**25f**) in a small amount of DMF (roughly 0.2 mL) was heated at 65 °C for 3 h under inert atmosphere in the dark. After that, the crude reaction mixture was concentrated to dryness under reduced pressure, and the residue was purified by column chromatography (cyclohexane → 3:1 cyclohexane-EtOAc) to give compounds **24**, **25**.

*O*-[2-(4′-Hydroxyphenyl)ethyl]-*N*-phenylselenocarbamate (**24**). Phenyl isoselenocyanate **12a** (53 mg, 0.29 mmol) was used. Compound **24** was obtained as an amorphous solid. Yield: 24 mg, 40%; *R*_F_ 0.48 (1:2 EtOAc-hexane); ^1^H-NMR (300 MHz, CD_3_OD) δ 7.25 (m, 2H, Ar-H*m*, Ph), 7.20–7.05 (m, 3H, Ar-H*o*, Ar-H*p*, Ph), 7.03 (m, 2H, Ar-H*o*, ArOH), 6.71 (m, 2H, Ar-H*m*, ArOH), 4.81 (t, 2H, *J*_H,H_ = 6.6 Hz, CH_2_O), 2.98 (t, 2H, CH_2_Ar) ppm; ^13^C-NMR (75.5 MHz, CD_3_OD) δ 191.7 (CSe), 157.1 (Ar-C*p*, ArOH), 138.8 (Ar-C*ipso*, Ph), 131.0 (Ar-C*o* ArOH), 129.9 (Ar-C*o*, Ph), 129.7 (Ar-C*ipso*, ArOH), 126.5 (Ar-C*p*, Ph), 123.4 (Ar-C*m*, Ph), 116.3 (Ar-C*m*, ArOH), 77.3 (CH_2_O), 35.0 (CH_2_Ar) ppm; HRCI-MS *m/z* calcd for C_15_H_15_NO_2_^80^Se ([M]^+^): 321.0263, found: 321.0270.

*O*-[2-(3′,4′-Dihydroxyphenyl)ethyl]-*N*-phenylselenocarbamate (**25a**). Phenyl isoselenocyanate **12a** (53 mg, 0.29 mmol) was used. Compound **25a** was obtained as a yellow solid. Yield: 44 mg, 69%; mp: 116–118 °C (MeOH); *R*_F_ 0.26 (1:2 EtOAc-hexane); ^1^H-NMR (300 MHz, CDCl_3_) δ 9.09 (brs, 1H, NH), 7.24 (m, 2H, Ar-H*m*, Ph), 7.20–7.05 (m, 3H, Ar-H*o*, Ar-H*p*, Ph), 6.80 (d, 1H, *J*_5,6_ = 8.0 Hz, H-5, HTyr), 6.71 (brs, 1H, H-2, HTyr), 6.61 (brd, 1H, *J*_5,6_ = 8.0 Hz, H-6, HTyr), 4.83 (t, 2H, *J*_H,H_ = 6.6 Hz, CH_2_O), 2.97 (t, 2H, CH_2_Ar) ppm; ^13^C-NMR (75.5 MHz, CDCl_3_) δ 190.9 (CSe), 143.7, 142.5 (C-3, C-4, HTyr), 136.6 (Ar-C*ipso*, Ph), 129.9 (C-1, HTyr), 129.1 (Ar-C*o*, Ph), 126.0 (Ar-C*p*, Ph), 122.0 (Ar-C*m*, Ph), 121.4 (C-6, HTyr), 116.0 (C-2, HTyr), 115.5 (C-5, HTyr), 77.3 (CH_2_O), 34.2 (*C*H_2_Ar); HRCIMS *m/z* calcd for C_15_H_15_NO_3_^80^Se ([M]^+^): 337.0212, found: 337.0217.

*O*-[2-(3′,4′-Dihydroxyphenyl)ethyl]-*N*-[4-methoxyphenyl]selenocarbamate (**25b**). 4-Methoxyphenyl isoselenocyanate **12b** (51 mg, 0.24 mmol) was used. Compound **25b** was obtained as s sticky solid. Yield: 9.7 mg; 14%; *R_F_* 0.24 (1:1 cyclohexane-EtOAc). ^1^H-NMR (500 MHz, CD_3_OD, −20 °C) Major isomer: δ 6.97 (m, 2H, ArH-*m*, Ph), 6.79 (m, 4H, 2 ArH-*o*, Ph), 6.70 (m, 1H, H-5, HTyr), 6.67 (d, 1H, *J*_2,6_ = 2.1 Hz, H-2, HTyr), 6.56 (dd, 1H, *J*_5,6_ = 8.0 Hz H-6, HTyr), 4.76 (t, 2H, *J*_H,H_ = 6.6 Hz, OCH_2_), 3.77 (s, 3H, OMe), 2.91 (t, 2H, Ar*C*H_2_) ppm; minor isomer: δ 7.43 (m, 2H, ArH-*m*, Ph), 6.90 (m, 4H, 2 ArH-*o*, Ph) ppm; ^13^C-NMR (125.7 MHz, CD_3_OD, −20 °C) δ 190.6 (CSe), 158.6 (ArC-*p*), 146.3 (C-3, HTyr), 144.9 (C-4, HTyr), 131.8 (Ar-C-*ipso*, Ph), 130.4 (C-1, HTyr), 124.8 (2 ArC-*o*, Ph), 121.1 (C-6, HTyr), 117.0 (C-2, HTyr), 116.2 (C-5, HTyr), 114.7 (ArC-*m*, Ph), 77.1 (OCH_2_), 55.7 (OMe), 35.2 (ArCH_2_) ppm; HRESI-MS *m/z* calcd for C_16_H_17_NNaO_4_^80^Se ([M+Na]^+^): 390.0215, found: 390.0209.

*N*-[4-Chlorophenyl]-*O*-[2-(3′,4′-dihydroxyphenyl)ethyl]selenocarbamate (**25c**). 4-Chlorophenyl isoselenocyanate **12c** (51.5 mg, 0.24 mmol) was used. Compound **25c** was obtained as a colouress sticky solid. Yield: 40.9 mg, 58%; *R_F_* 0.54 (1:1 cyclohexane-EtOAc). ^1^H-NMR (500 MHz, CD_3_OD, −20 °C) δ 7.22 (m, 2H, Ar-H*m*), 7.03 (m, 2H, Ar-H*o*), 6.70 (m, 3H, H-2, H-5, H-6, catechol), 4.81 (t, 2H, *J*_H,H_ = 6.6 Hz, OCH_2_), 2.95 (t, 2H, ArC*H*_2_) ppm; ^13^C-NMR (125.7 MHz, CD_3_OD, −20 °C) δ 191.6 (CSe), 146.4, 145.1 (ArC-3, ArC-4, catechol), 137.6 (Ar-C*ipso*), 131.1 (C-1, catechol), 130.4 (Ar-C*p*), 129.9 (2 Ar-C*m*), 124.4 (2 Ar-C*o*), 121.1 (C-6, catechol), 116.9, 116.3 (C-2, C-5, catechol), 77.4 (OCH_2_), 35.0 (Ar*C*H_2_) ppm; HRESI-MS *m/z* calcd for C_15_H_14_ClNNaO_3_^80^Se ([M + Na]^+^): 393.9720, found: 393.9712.

*N*-[4-Bromophenyl]-*O*-[2-(3′,4′-dihydroxyphenyl)ethyl]selenocarbamate (**25d**). 4-Bromophenyl isoselenocyanate **12d** (62.1 mg, 0.24 mmol) was used. Compound **25d** was obtained as a colouress sticky solid. Yield: 74.1 mg, 94%; *R_F_* 0.56 (1:1 cyclohexane-EtOAc). ^1^H-NMR (500 MHz, CD_3_OD, −20 °C) δ 7.63, 7.36 (m, 4H, 2 ArH-*m*), 7.49 (m, 2H, Ar-H*m*). 6.96 (m, 2H, Ar-H*o*), 6.72 (d, 1H, *J*_5,6_ = 8.0 Hz, H-5, catechol), 6.70 (d, 1H, *J*_2,6_ = 2.1 Hz, ArH-2), 6.59 (dd, 1H, ArH-6), 4.80 (t, 2H, *J*_H,H_ = 6.7 Hz, OCH_2_), 2.94 (t, 2H, ArC*H*_2_) ppm; ^13^C-NMR (125.7 MHz, CD_3_OD, −20 °C) δ 191.5 (CSe), 146.4 rC-3, catechol), 145.1 (C-4, catechol), 138.1 (Ar-C*ipso*), 132.9 (Ar-C*m*), 132.6 (C-1, catechol), 130.4 (Ar-C*p*), 124.6 (Ar-C*o*), 121.1 (C-6, catechol), 116.9 (C-2, catechol), 116.3 (C-5, catechol), 77.5 (OCH_2_), 35.0 (Ar*C*H_2_) ppm; HRESI-MS *m/z* calcd for C_15_H_14_^79^BrNNaO_3_^80^Se [M + Na]^+^: 437.9214, found: 437.9205; HRESI-MS *m/z* calcd for C_15_H_14_^81^BrNNaO_3_^80^Se ([M + Na]^+^): 439.9194, found: 439.9190.

*O*-[2-(3′,4′-Dihydroxyphenyl)ethyl]-*N*-[4-iodophenyl]selenocarbamate (**25e**). 4-Iodophenyl isoselenocyanate **12e** (73.3 mg, 0.24 mmol) was used. Compound **25e** was obtained as a sticky solid. Yield: 79.9 mg, 91%; *R_F_* 0.55 (1:1 cyclohexane-EtOAc). ^1^H-NMR (500 MHz, CD_3_OD, −20 °C) δ 7.55 (m, 2H, Ar-H*m*), 6.85 (m, 2H, Ar-*Ho*), 6.72 (d, 1H, *J*_5,6_ = 8.1 Hz, H-5, catechol), 6.71 (d, 1H, *J*_2,6_ = 2.0 Hz, H-2, catechol), 6.59 (dd, 1H, H-6, catechol), 4.80 (t, 2H, *J*_H,H_ = 6.6 Hz, OCH_2_), 2.95 (t, 2H, ArC*H*_2_) ppm; ^13^C-NMR (125.7 MHz, −20 °C, CD_3_OD) δ 191.5 (CSe), 146.4 (C3, catechol), 145.0 (C-4, catechol), 139.0 (Ar-C*m*), 138.6 (Ar-C*ipso*), 130.4 (C-1, catechol), 124.7 (Ar-C*o*), 121.06 (C-6, catechol), 116.9 (C-2, catechol), 116.2 (C-5, catechol), 89.6 (Ar-C*p*), 77.5 (OCH_2_), 35.0 (CH_2_) ppm; HRESI-MS *m/z* calcd for C_15_H_14_INNaO_3_^80^Se ([M + Na]^+^): 485.9076, found: 485.9064.

*O*-[2-(3′,4′-Dihydroxyphenyl)ethyl]-*N*-[1,1’-biphenyl]-4-yl]selenocarbamate (**25f**). (1,1’-Biphenyl]-4-yl isoselenocyanate **12f** (61.6 mg, 0.238 mmol) was used. Compound **25f** was obtained as a sticky solid. Yield: 25.0 mg, 32%; *R_F_* 0.53 (1:1 cyclohexane-EtOAc). ^1^H-NMR (500 MHz, CD_3_OD, −20 °C) δ 7.62 (m, 2H, PhH-*m*), 7.51 (m, 2H, ArH-*m*), 7.44 (m, 2H, PhH-*o*), 7.34 (m, 1H, PhH-*p*), 7.14 (m, 2H, ArH-*o*), 6.73 (d, 1H, *J*_5,6_ = 8.1 Hz, H-5, catechol), 6.73 (d, 1H, *J*_2,6_ = 2.1 Hz, H-2, catechol), 6.62 (dd, 1H, H-6, catechol), 4.83 (t, 2H, *J*_H,H_ = 6.7 Hz, OCH_2_), 2.97 (t, 2H, ArC*H*_2_) ppm; ^13^C NMR (125.7 MHz, CD_3_OD, −20 °C) δ 191.3 (CSe), 146.4, 145.1 (C-3, C-4, catechol), 141.5 (Ar-C*ipso*), 139.1 (PhC-*ipso*), 138.2 (Ar-C*p*), 130.5 (C-1, catechol), 129.9 (PhC-*m*), 128.3 (Ar-C*m*), 127.8 (PhC-*o*), 125.7 (PhC-*p*), 123.3 (Ar-C*o*), 121.1 (C-5, catechol), 117.1 (C-2, catechol), 116.3 (C-6, catechol), 77.4 (OCH_2_), 35.1 (Ar*C*H_2_) ppm; HRESI-MS *m/z* calcd for C_21_H_19_NNaO_3_^80^Se ([M + Na]^+^): 436.0422, found: 436.0416.

#### 3.3.4. General Procedure for the Preparation of Selenocarbamates **26**, **27**

A mixture of tyrosol **22** (60 mg, 0,43 mmol, 1.0 equiv.) or hydroxytyrosol **23** (66 mg, 0.43 mmol, 1.0 equiv.) and α-naphthyl isoselenocyanate **13** (100 mg, 0.43 mmol, 1.0 equiv.) in a small amount of DMF (roughly 0.2 mL) was heated at 80 °C during 7 h. Then, it was concentrated to dryness under reduced pressure, and the residue was purified by column chromatography (hexane → 3:1 hexane-EtOAc) to give **26** and **27** as solids.

*O*-[2-(4-Hydroxyphenyl)ethyl]-*N-*(α-naphthyl)selenocarbamate (**26**). Yield: 88 mg, 55%; mp: 128–130 °C (MeOH); *R*_F_ 0.62 (1:2 EtOAc-hexane); ^1^H-NMR (300 MHz, CDCl_3_) δ 9.21 (brs, 1H, NH), 7.90–7.80 (m, 3H, H-4, H-6, H-7, naphthyl), 7.54–7.49 (m, 2H, H-5, H-8, naphthyl), 7.40 (t, 1H, H-3, naphthyl), 7.24 (d, 1H, *J*_2,3_ = 7.8 Hz, H-2, naphthyl), 6.71 (m, 2H, H-2, H-6, Ar-OH), 6.57 (m, 2H, H-3, H-5, Ar-OH), 5.22 (brs, 1H, OH), 4.72 (t, 2H, *J*_H,H_ = 6.6 Hz, CH_2_O), 2.79 (t, 2H, CH_2_Ar) ppm; ^13^C-NMR (75.5 MHz, CDCl_3_) δ 192.8 (CSe), 154.3 (C-4, Tyr), 134.2 (C-4a, Naph), 132.3, 130.1 (C-1, C-2, C-6, Tyr), 129.3, 128.5, 128.2, 127.2, 126.7, 125.4, 123.7, 122.2 (C-2, C-3, C-4, C-5, C-6, C-7, C-8, C-8a, naphthyl), 115.3 (C-3, C-5, Tyr), 76.9 (CH_2_O), 34.0 (CH_2_Ar) ppm; HRLSI-MS *m/z* calcd. for C_19_H_17_NNaO_2_^80^Se ([M + Na]^+^): 394.0317, found: 394.0331.

*O*-[2-(3′,4′-Dihydroxyphenyl)ethyl]-*N-*(α-naphthyl)selenocarbamate (**27**). Yield: 106 mg, 64%; mp: 50–52 °C (CH_2_Cl_2_/MeOH); *R*_F_ 0.18 (1:2 EtOAc-hexane 1:2); ^1^H-NMR (500 MHz, CDCl_3_) δ 9.12 (brs, 1H, NH), 7.89–7.81 (m, 3H, H-4, H-6, H-7, naphthyl), 7.54–7.47 (m, 2H, H-5, H-8, naphthyl), 7.42 (t, 1H, H-3, naphthyl), 7.27 (d, 1H, *J*_2,3_ = 7.8 Hz, H-2, naphthyl), 6.61 (d, 1H, *J*_5,6_ = 7.7 Hz, H-5), 6.32 (brd, 1H, H-6, catechol), 6.30 (brs, 1H, H-2, catechol), 5.45–5.20 (brs, 2H, OH), 4.71 (t, 2H, *J*_H-H_ = 6.4 Hz, CH_2_O), 2.73 (t, 2H, CH_2_Ar) ppm; ^13^C-NMR (125.7 MHz, CDCl_3_) δ 192.9 (CSe), 143.3, 142.4 (C-3, C-4, catechol), 134.2 (C-4a, naphthyl), 132.4 (C-1, catechol), 130.1, 128.6, 128.5, 128.2, 127.3, 126.8, 125.5, 123.8, 122.2, 121.6 (C-2, C-3, C-4, C-5, C-6, C-7, C-8, C-8a, naphthyl; C-6, catechol), 116.1 (C-2, catechol), 115.4 (C-5, catechol), 76.7 (CH_2_O), 34.2 (CH_2_Ar) ppm; HRCI-MS *m/z* calcd for C_19_H_18_NO_3_^80^Se ([M + H]^+^): 388.0446, found: 388.0455.

## 4. Conclusions

Herein, we have accomplished the design and chemoselective synthesis of three different families of organoselenium-polyphenolic hybrids with the aim of developing novel antioxidant and antiproliferative agents: carbohydrate-containing *N*-acyl,*Se*-phenacyl isoselenoureas, *N*-aryl,*Se*-phenacyl isoselenocarbamates and *N*-aryl,*O*-phenetyl selenocarbamates. These compounds were subjected to the following biological assays: antiradical (DPPH method), DNA-protective effects, GPx-like properties, and antiproliferative assay towards a panel of six human tumour cell lines. The nature of the organoselenium motif, number and position of phenolic hydroxyl groups and substituents on the aromatic scaffolds afforded a series of valuable structure–activity relationships. Particularly relevant are *O*-phenetyl selenocarbamates **24**–**27**, which behaved as excellent mimetics of glutathione peroxidase (GPx); when used in the substoichiometric ratio, they efficiently eliminated H_2_O_2_ used herein as an ROS model. Regarding antiproliferative activities, prepared isoselenocarbamates and selenocarbamates behaved as potent antiproliferative activities (low micromolar range) against non-small lung cell lines (A549, SW1573), with similar potency as that shown by the chemotherapeutic agent cisplatin (CDDP), and occasionally, with potency higher than VP-16.

Therefore, the hybridization of organoselenium/phenolic scaffolds seems to be a useful approach for the development of potential anticancer agents. In particular, selenocarbamates **24**–**27** could be the start point of a new future generation of antiproliferative agents that can be endowed with dual activity—a preventive action, due to their excellent DPPH and GPx-mimicry activities, thus lowering the levels of hazardous ROS. This might diminish the probability of the development of tumour processes. Conversely, they could also be used as effective chemotherapeutic agents against cancer, as a result of their good antiproliferative properties. 

## Figures and Tables

**Table 1:** Antioxidant assays for organoselenium derivatives.

Compound		DPPH (EC_50_, µM) ^c^	%DNA Oxidation Inhibition ^a,c^
Selenourea	**3**	22 ± 1	9.2 ± 3.5
Isoselenoureas	**7**	>250	8.2 ± 2.2
	**8**	>250	3.8 ± 5.6
	**9**	15 ± 1	**32 ± 2**
*O*-Phenacyl isoselenocarbamates	**16**	>250	1.9 ± 0.20
	**17**	>250	6.5 ± 3.9
	**18**	**8.0 ± 0.2**	**38 ± 5**
	**19**	>250	9.1 ± 0.20
	**20**	>250	1.3 ± 1.7
	**21**	**7.8 ± 0.3**	**31 ± 5**
Selenocarbamates	**24**	64 ± 2.0	9.4 ± 5.0
	**25a**	12 ± 1	**33 ± 1**
	**25b**	16 ± 3	N.T. ^b^
	**25c**	**6.3 ± 0.8**	N.T.^b^
	**25d**	**4.7 ± 0.5**	N.T.^b^
	**25e**	**4.8 ± 0.3**	N.T.^b^
	**25f**	**5.2 ± 0.2**	N.T.^b^
	**26**	71 ± 1	**29 ± 9**
	**27**	15 ± 1	**32 ± 1**
Controls	**22 (Tyr)**	>500	18 ± 1
	**23 (HTyr)**	15 ± 2	41 ± 3
	**BHT** [45]	70 ± 9	4.1 ± 1.0

^a^ at 0.5 mM; ^b^ not tested; ^c^ Bold data represent the strongest compounds.

**Table 2:** Glutahione peroxidase mimicry of seleno-derivatives ^a^.

Compound ^b^		t_1/2_ (min)
Selenourea	**3**	5.9
Isoselenoureas	**7**	3.4
	**8**	5.9
	**9**	No activity
*O*-Phenacyl isoselenocarbamates	**16**	233
	**17**	7.1
	**18**	2.8
	**19**	No activity
	**20**	4.9
	**21**	3.4
Selenocarbamates	**24**	<0.5 5.0 ^c^
	**25a**	1.0 8.7 ^c^
	**26**	2.9 18.3 ^c^
	**27**	0.9 9.4 ^c^
Control	HTyr	No activity

^a^ [DTT] = 0.27 mM, [H_2_O_2_] = 0.29 mM (initial concentrations); ^b^ at 5% molar concentration; ^c^ at 1% molar concentration.

**Table 3:** Antiproliferative activity of selenoderivatives (GI_50_, µM) ^a^.

Compound		A549 (Lung)	HBL-100 (Breast)	Hela (Cervix)	SW1573 (Lung)	T-47D (Breast)	WiDr (Colon)
Selenourea	**3**	**3.2 ± 0.84**	**3.5 ± 1.2**	**3.3 ± 1.4**	**4.4 ± 1.5**	**3.3 ± 0.20**	**4.9 ± 1.4**
Isoselenoureas	**8**	36 ± 3.3	39 ± 6.6	42 ± 9.5	35 ± 1.4	29 ± 5.6	45 ± 16
	**9**	>100	>100	>100	>100	>100	>100
Isoselenocarbamates	**16**	**8.6 ± 3.5**	26 ± 3.2	31 ± 3.1	31 ± 5.9	24 ± 4.4	45 ± 20
	**17**	**6.9 ± 1.3**	**10 ± 0.39**	14 ± 0.66	18 ± 4.8	**8.8 ± 3.7**	20 ± 6.2
	**19**	24 ± 6.1	30 ± 3.5	20 ± 5.5	90 ± 17	84 ± 11	34 ± 6.1
	**20**	19 ± 0.32	19 ± 4.2	15 ± 2.0	17 ± 3.5	18 ± 2.3	21 ± 2.9
	**21**	16 ± 0.62	16 ± 0.51	13 ± 1.6	14 ± 2.6	16 ± 1.0	18 ± 2.0
Selenocarbamates	**24**	13 ± 0.52	20 ± 1.8	15 ± 2.3	18 ± 5.1	23 ± 9.2	25 ± 6.1
	**25b**	**7.5 ± 1.5**	18 ± 0.21	19 ± 1.1	12 ± 1.7	17 ± 1.3	18 ± 1.2
	**25c**	**4.5 ± 1.8**	16 ± 2.6	13 ± 2.5	**5.9 ± 1.5**	20 ± 4.9	20 ± 7.0
	**25d**	**8.5 ± 4.4**	18 ± 2.7	15 ± 3.6	**6.5 ± 2.9**	21 ± 10	25 ± 11
	**25e**	**9.0 ± 3.8**	18 ± 3.1	14 ± 4.7	**6.6 ± 1.9**	23 ± 8.6	20 ± 5.5
	**25f**	11 ± 4.0	12 ± 2.7	**10 ± 2.7**	**3.7 ± 1.3**	16 ± 4.5	11 ± 2.0
	**26**	14 ± 1.8	18 ± 3.3	14 ± 1.4	15 ± 0.86	19 ± 1.1	22 ± 2.1
Polyphenols	Tyr	>100	>100	>100	>100	>100	>100
	HTyr	>100	82 ± 18	>100	50 ± 25	>100	>100
Control	VP-16	1.5 ± 0.25	1.2 ± 0.30	2.4 ± 0.94	15 ± 1.5	18 ± 4.4	24 ± 2.6
	CDDP	4.9 ± 0.2	1.9 ± 0.2	1.8 ± 0.5	2.7 ± 0.4	17 ± 3	26 ± 4

^a^ Bold data represent the strongest compounds.

## Data Availability

The data presented in this study are available on request from the corresponding author.

## Supplementary Materials

The following supporting information can be downloaded at: ^1^H- and ^13^C-NMR spectra.

## Sample Availability

Not available.
