# Supplementary material for: Chemoselective Preparation of New Families of Phenolic-Organoselenium Hybrids—A Biological Assessment
Source: Molecules. 2022 Feb 15;27(4):1315. doi: 10.3390/molecules27041315 (PMC8875169; doi:10.3390/molecules27041315)
Supplement: Supplementary file 1 [file molecules-27-01315-s001.zip › molecules-1548654-supplementary.pdf]

Chemoselective Preparation of New Families of Phenolic-Organoselenium Hybrids—A Biological Assessment

Paloma Begines [1], Sergio Martos [1], Irene Lagunes [2], Inés Maya [1], José M. Padrón [2,*], Óscar López [1,*] and José G. Fernández-Bolaños [1,*]

[1] Departamento de Química Orgánica, Facultad de Química, Universidad de Sevilla, Apartado 1203, E-41071 Seville, Spain.; pbegines@us.es (P.B.), smardel251@iespinorueda.com (S.M.), imaya@us.es (I.M.)

[2] BioLab, Instituto Universitario de Bio-Orgánica "Antonio González" (IUBO-AG), Universidad de La Laguna, c/ Astrofísico Francisco Sánchez 2, E-38206 La Laguna, Spain; roslagunes@uv.mx

* Correspondence: jmpadron@ull.es (J.M.P.), osc-lopez@us.es (O.L.), bolanos@us.es (J.G.F.-B.)

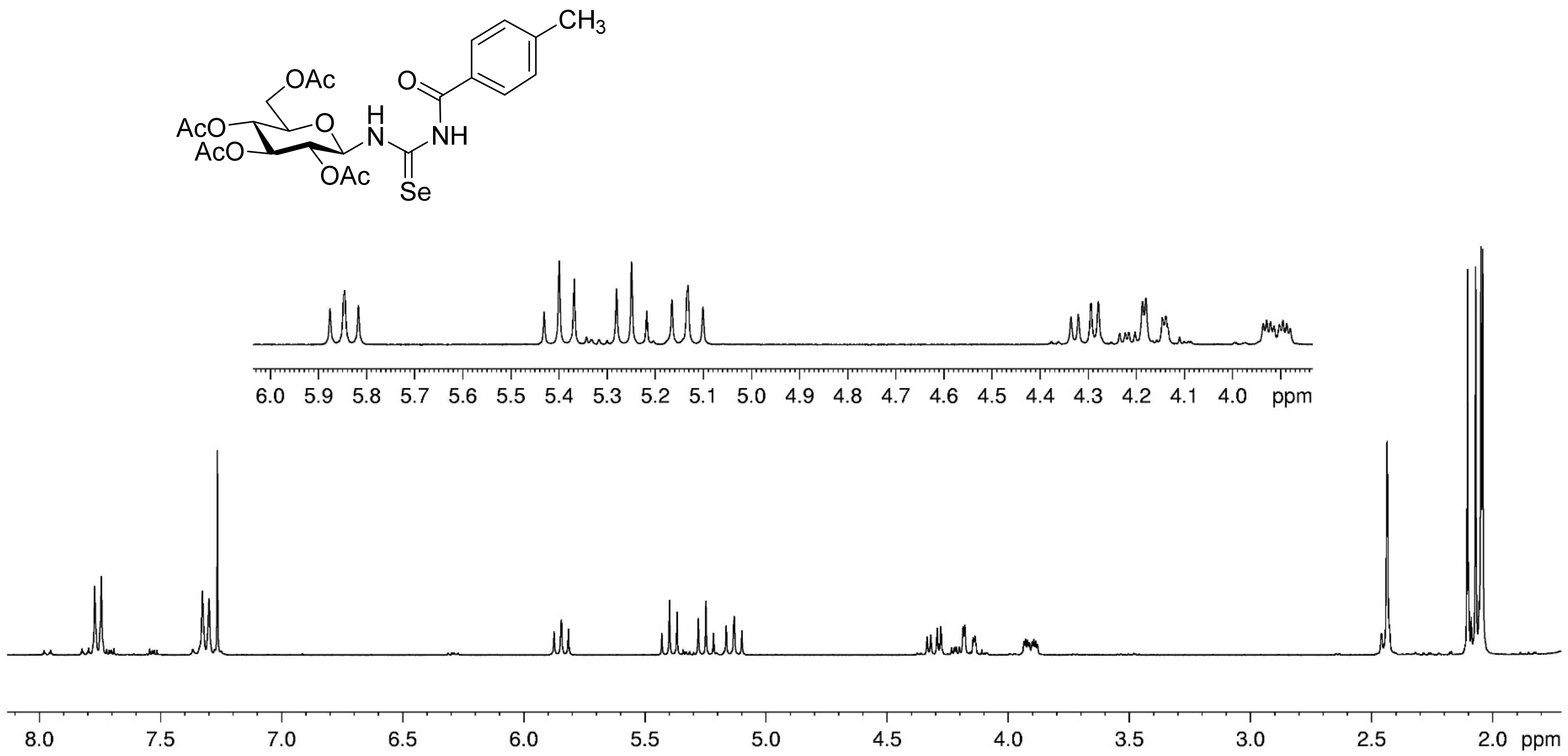

^{1}H-NMR (300 MHz, $CDCl_3$) of **3**

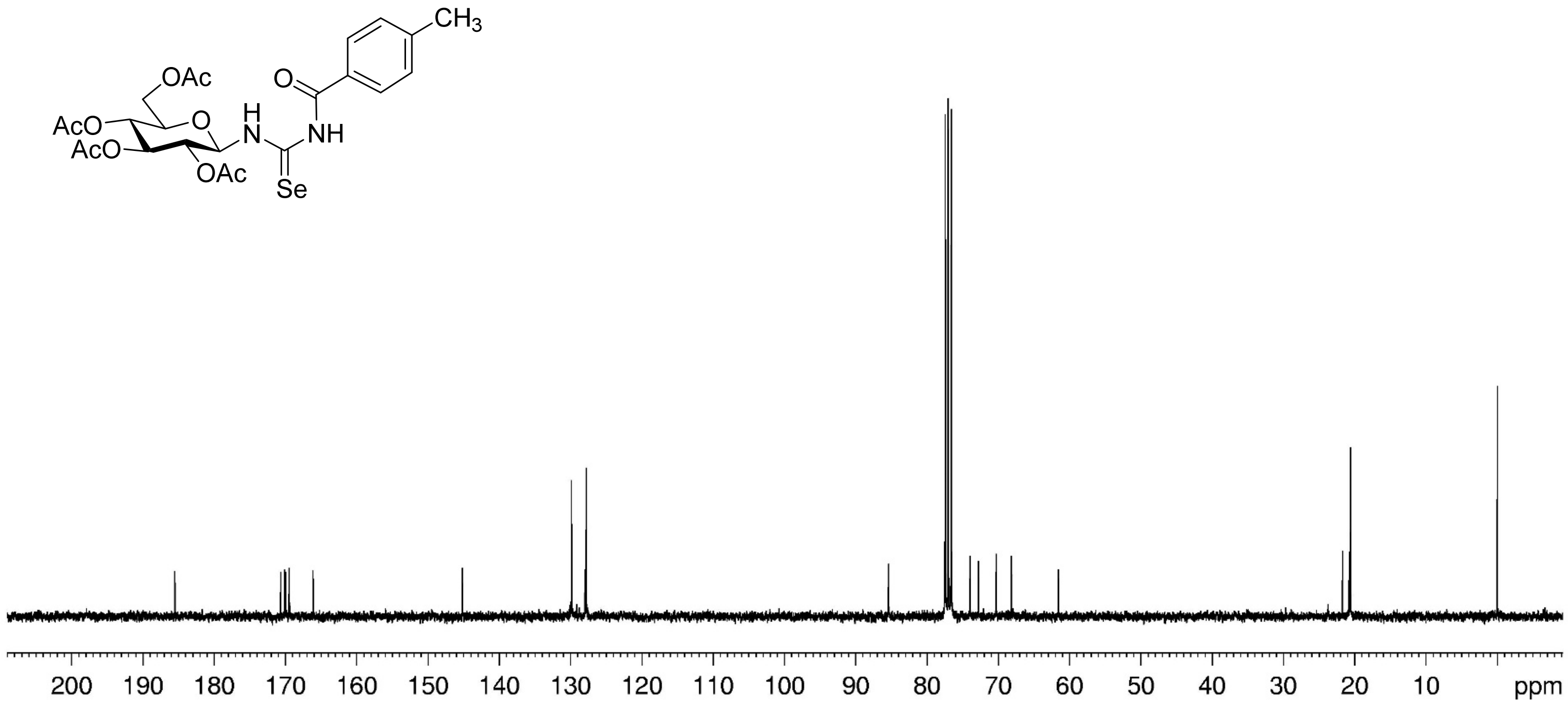

^{13}C-NMR (75.5 MHz, $CDCl_3$) of **3**

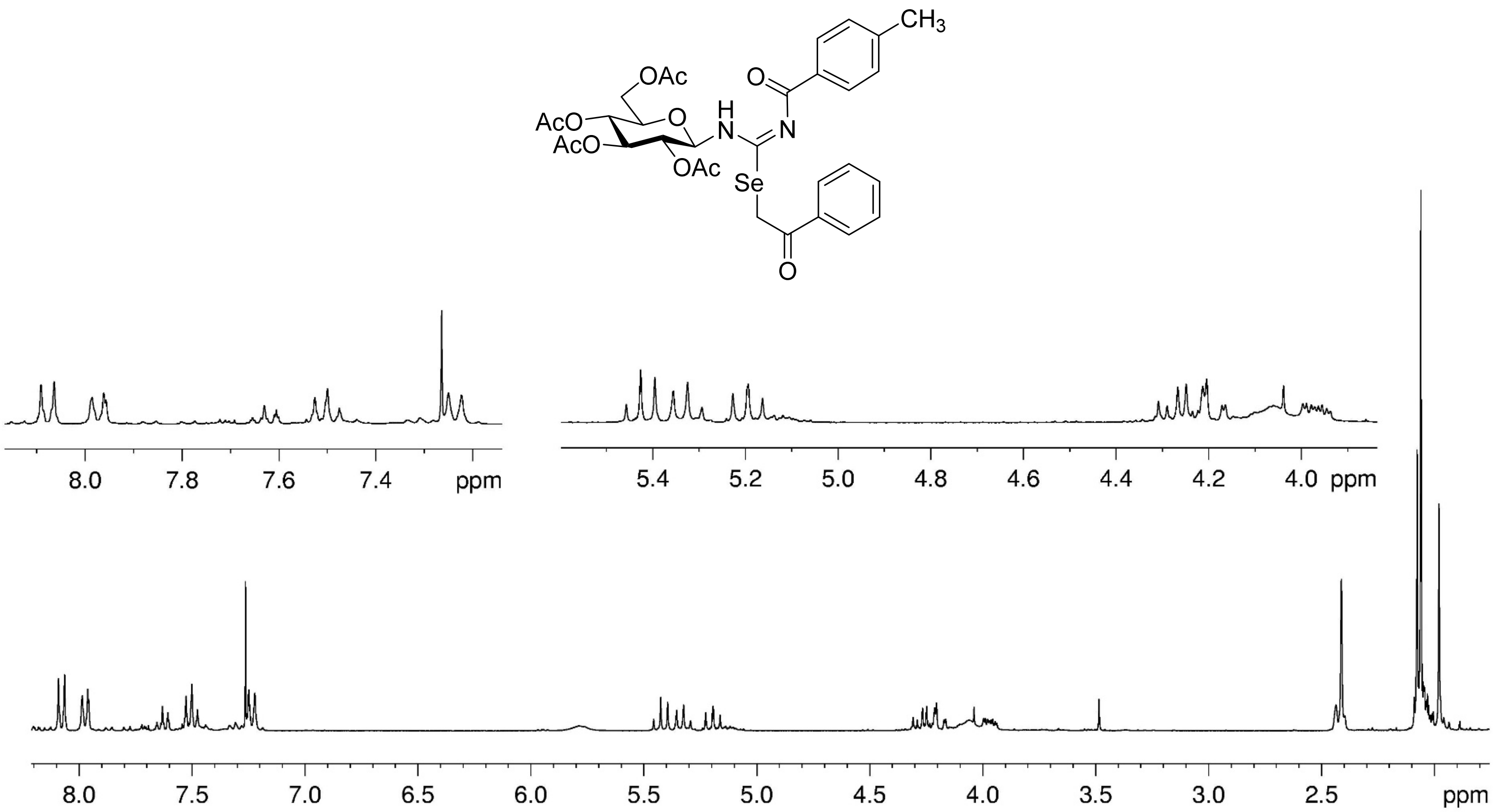

^{1}H-NMR (300 MHz, $CDCl_3$) of **7**

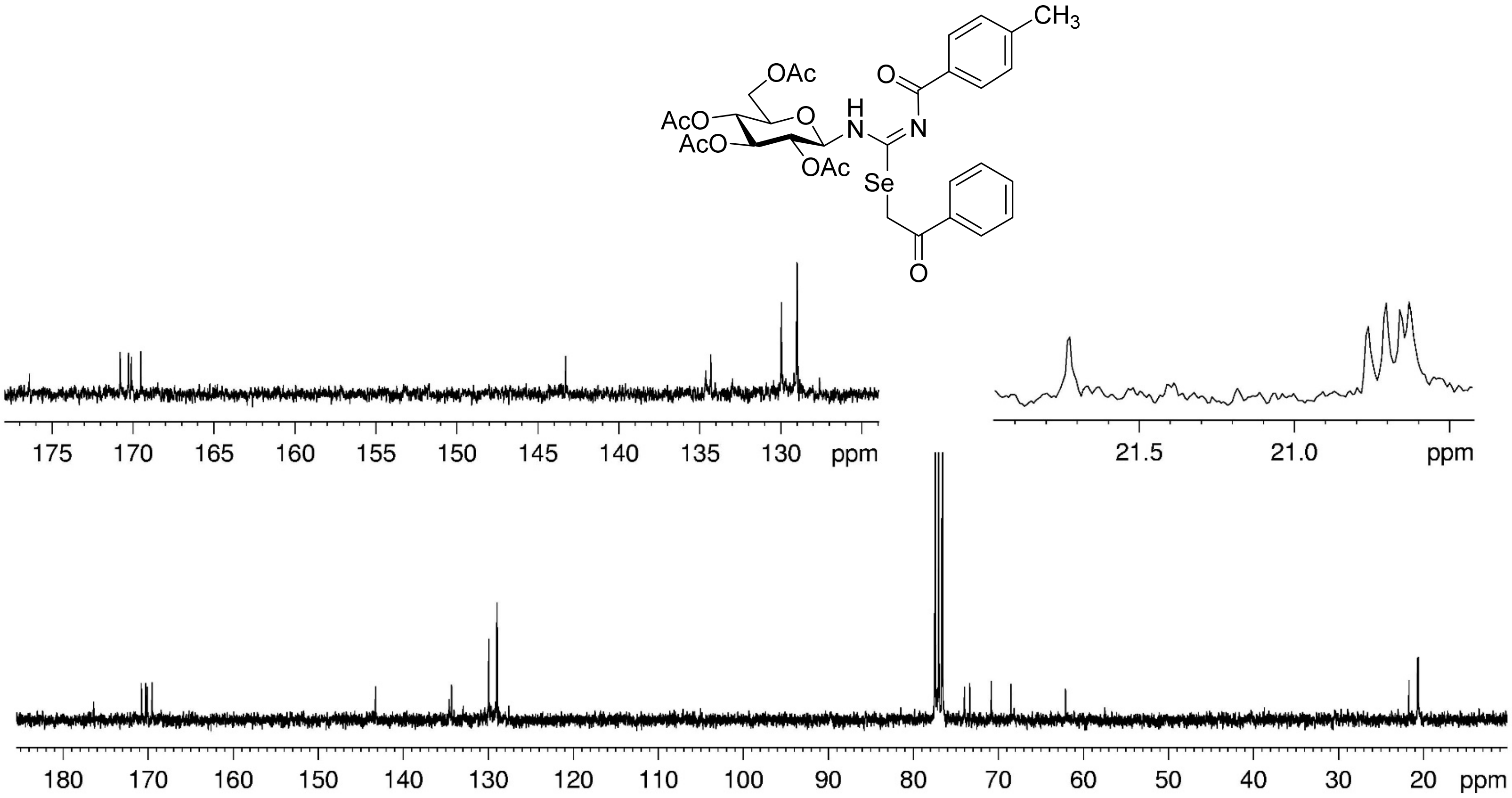

^{13}C-NMR (75.5 MHz, $CDCl_3$) of **7**

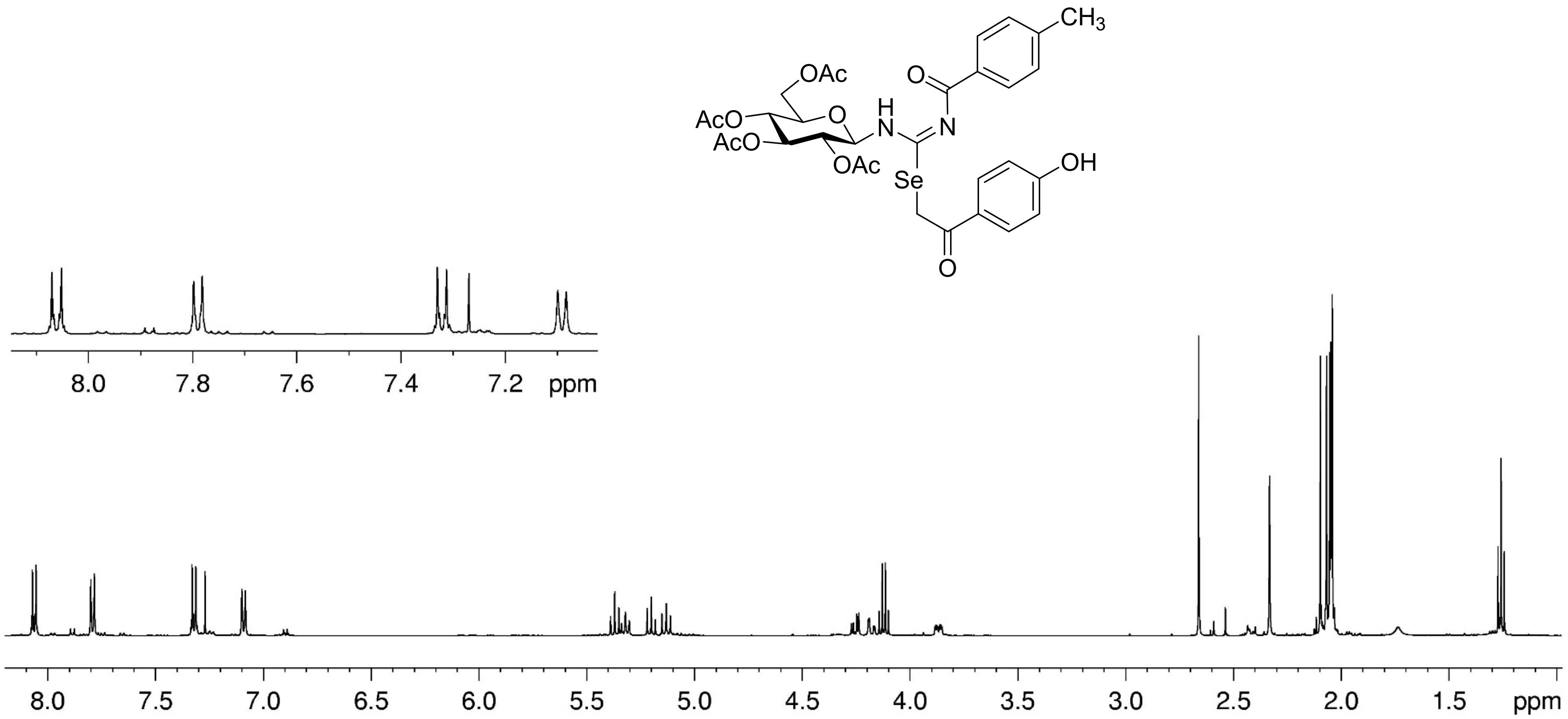

^{1}H-NMR (500 MHz, $CDCl_3$) of **8**

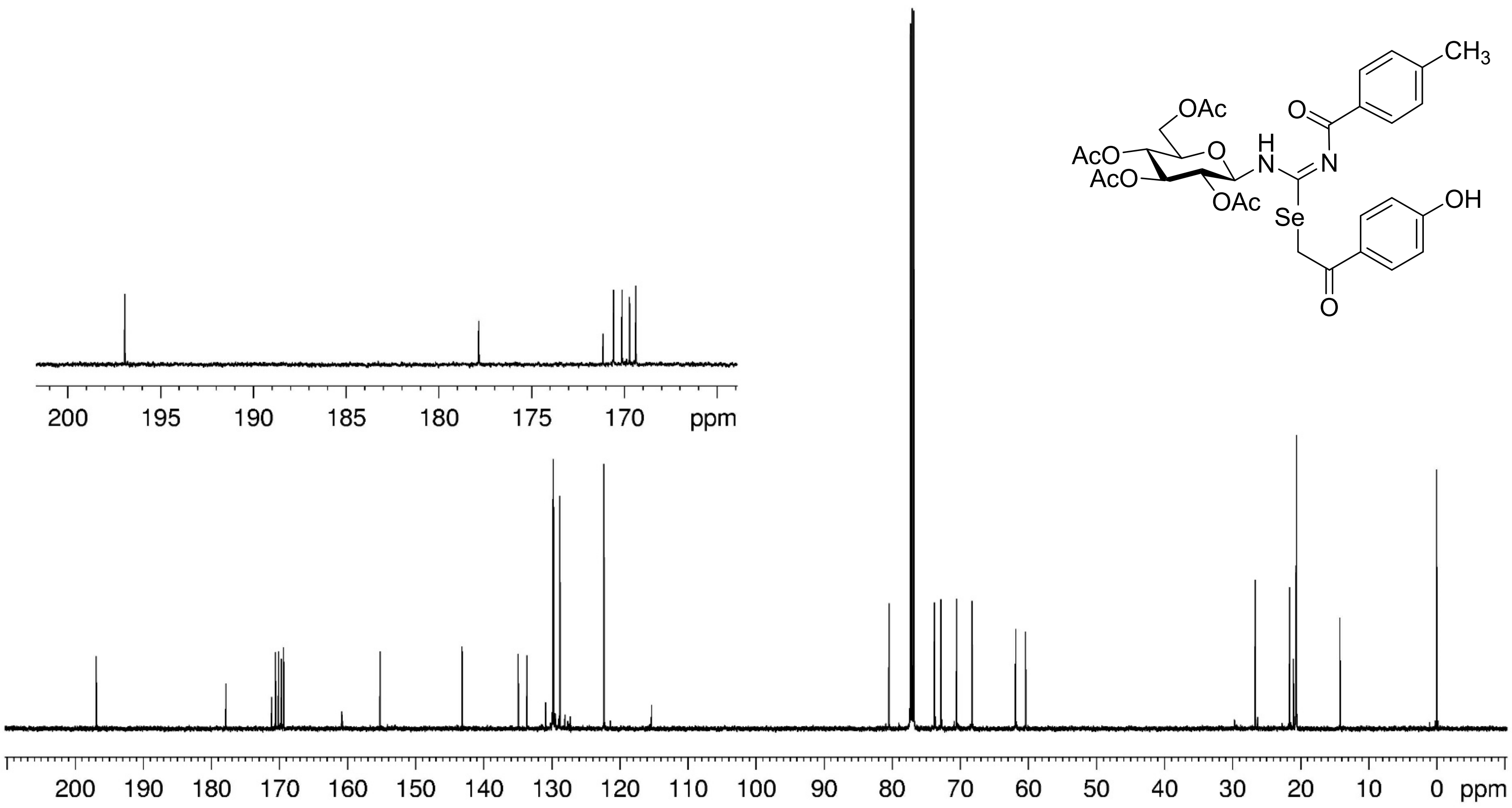

^{13}C-NMR (125.7 MHz, $CDCl_3$) of **8**

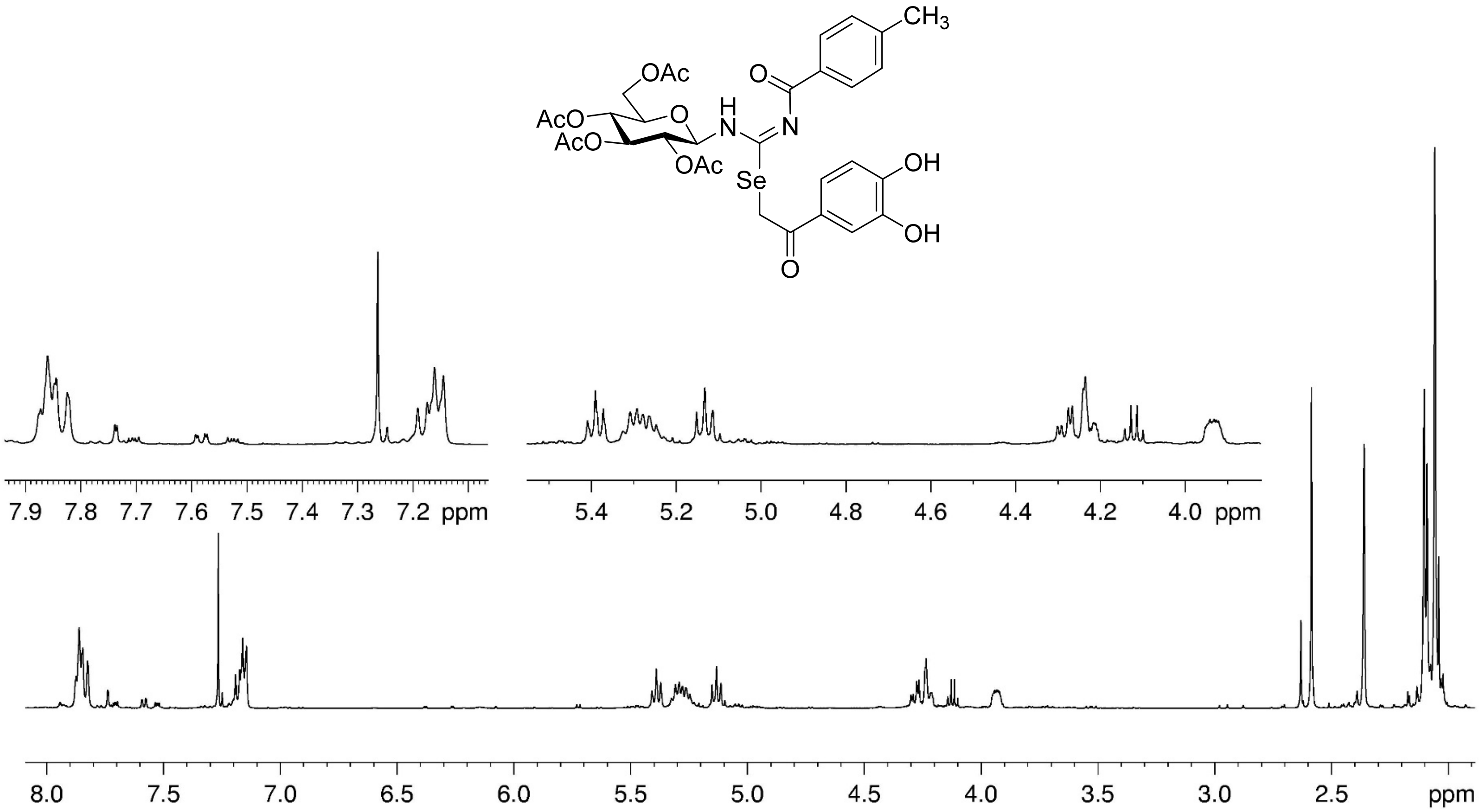

^{1}H-NMR (500 MHz, $CDCl_3$) of **9**

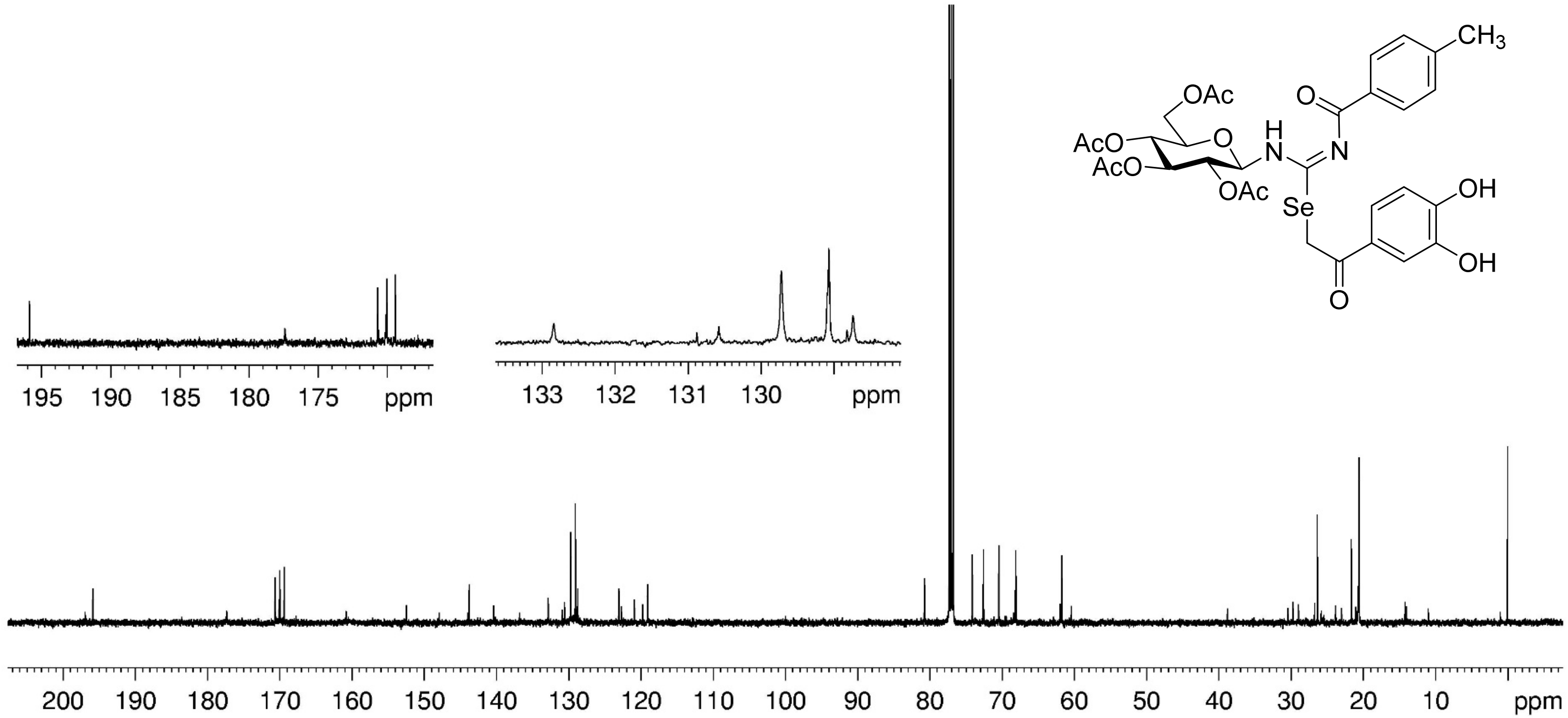

^{13}C-NMR (125.7 MHz, $CDCl_3$) of **9**

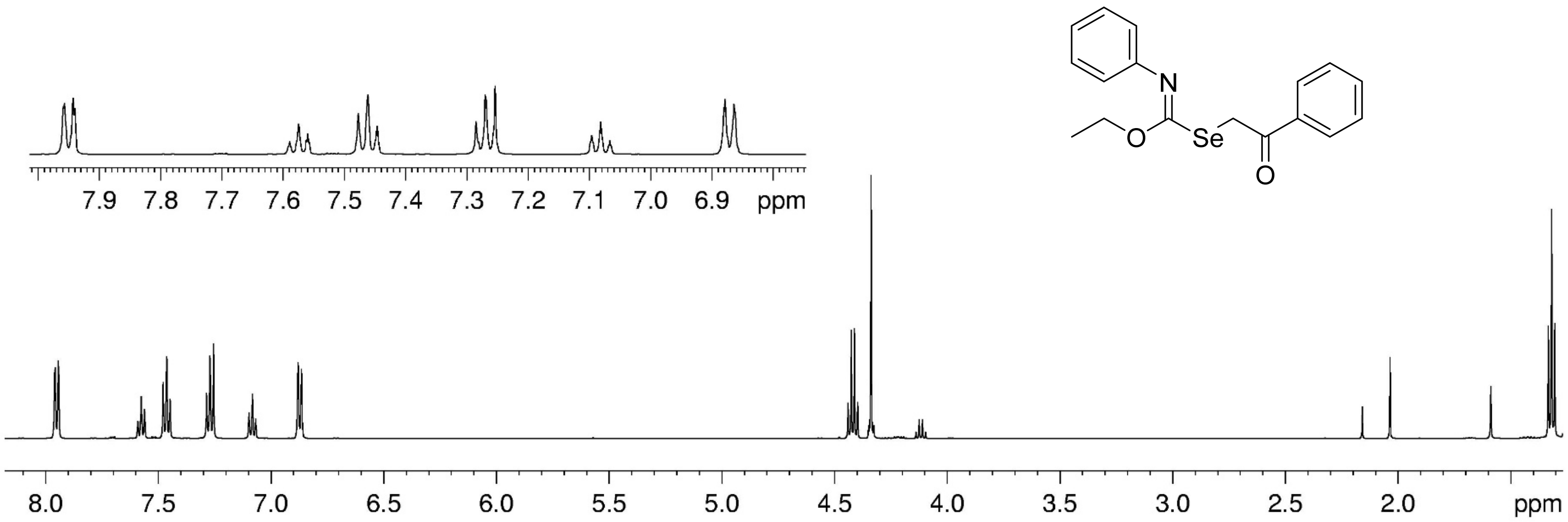

^{1}H-NMR (500 MHz, $CDCl_3$) of **16**

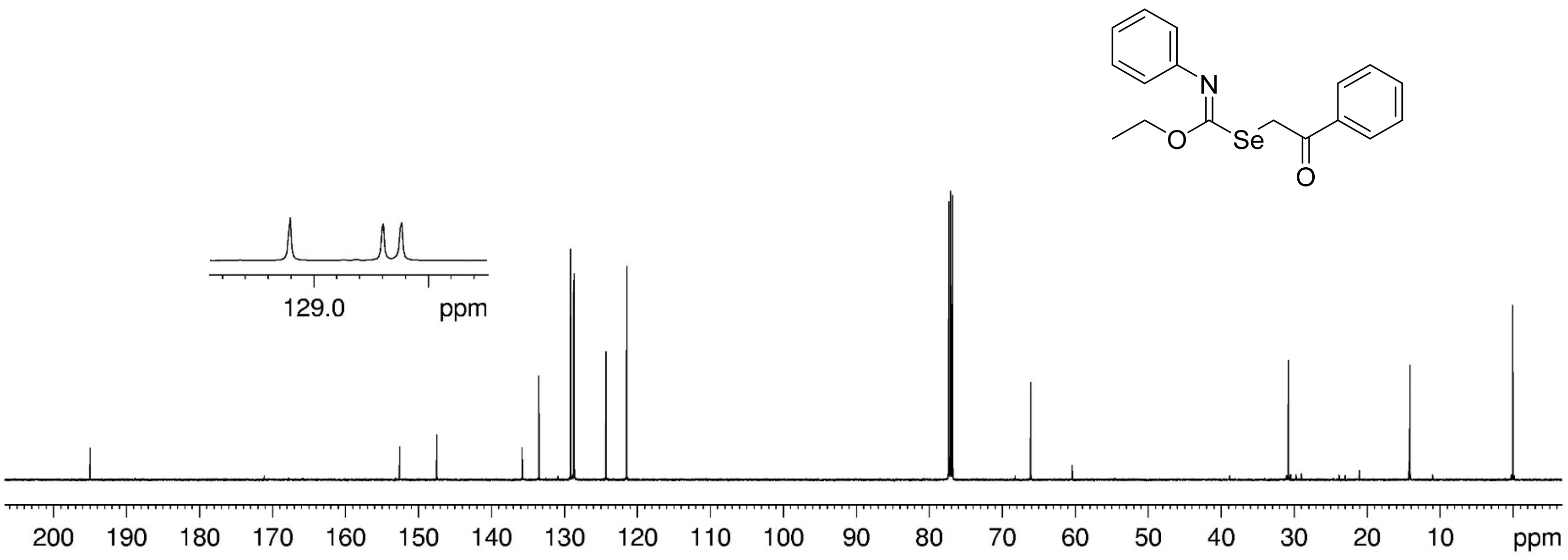

^{13}C-NMR (125.7 MHz, $CDCl_3$) of **16**

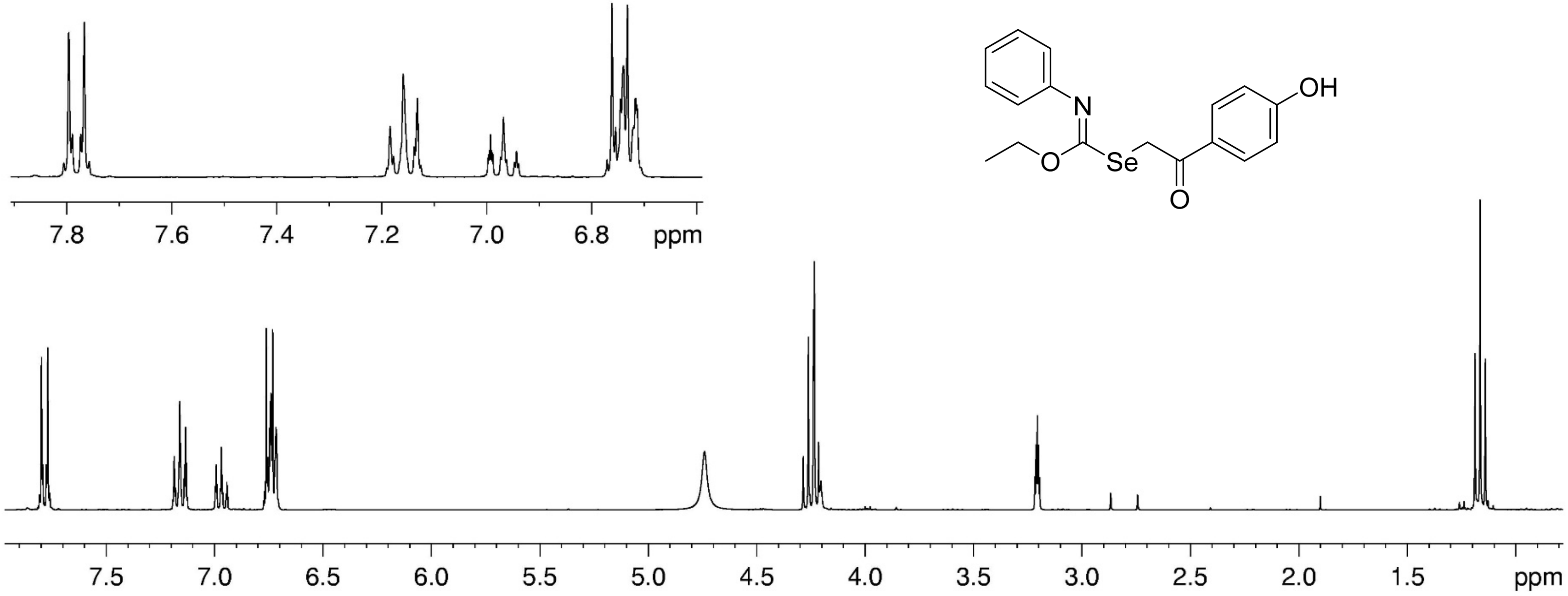

^{1}H-NMR (300 MHz, CD_3OD) of **17**

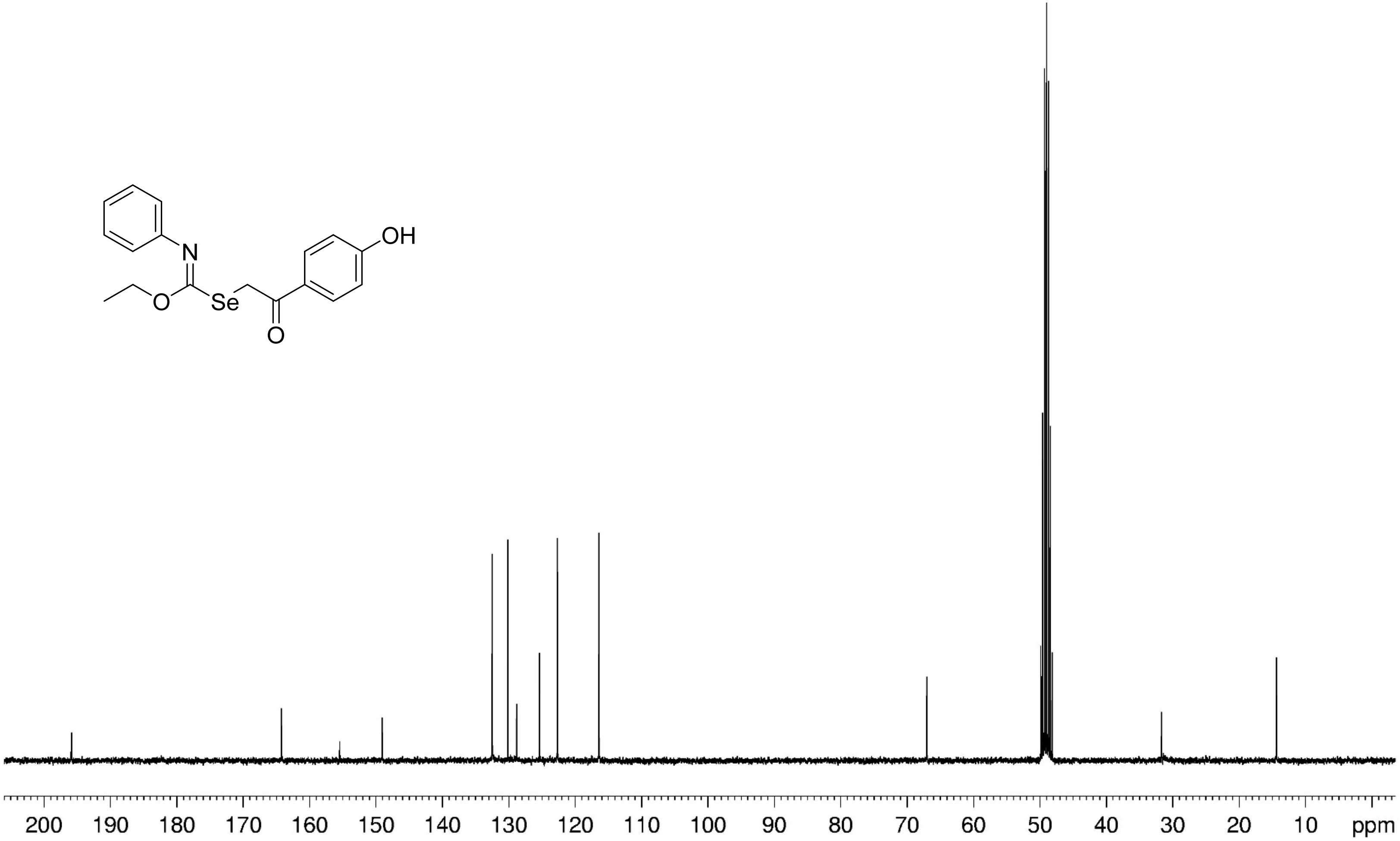

^{13}C-NMR (125.7 MHz, CD_3OD) of **17**

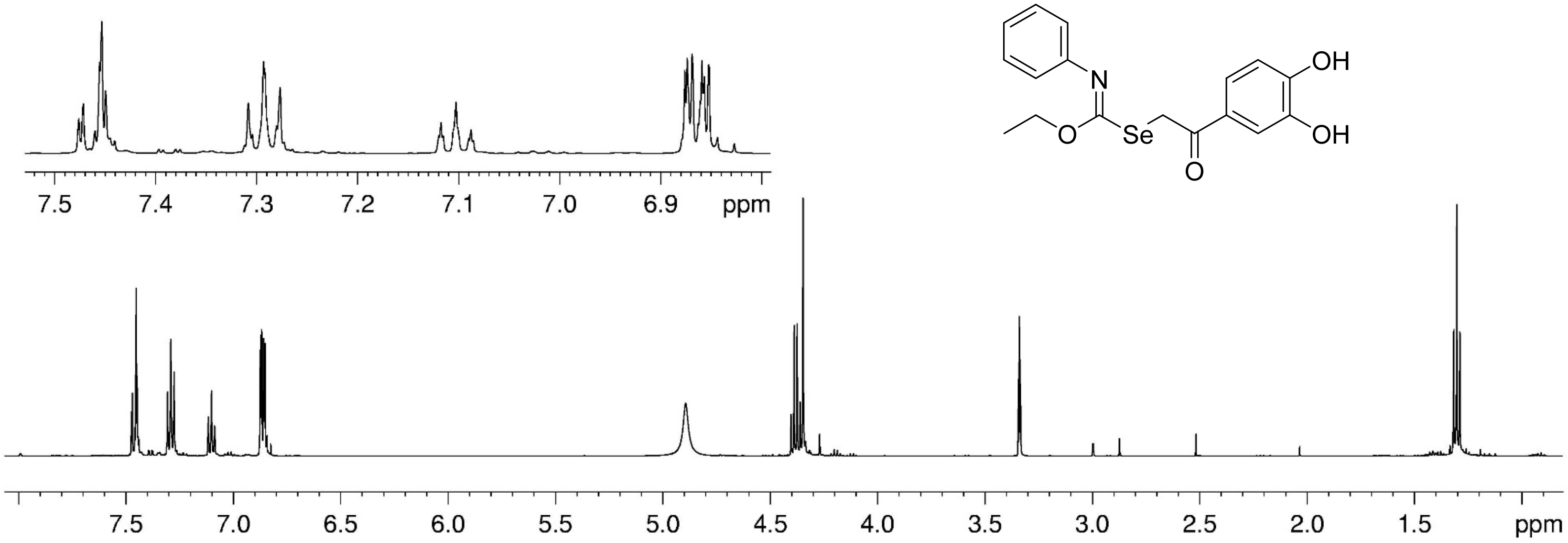

^{1}H-NMR (500 MHz, CD_3OD) of **18**

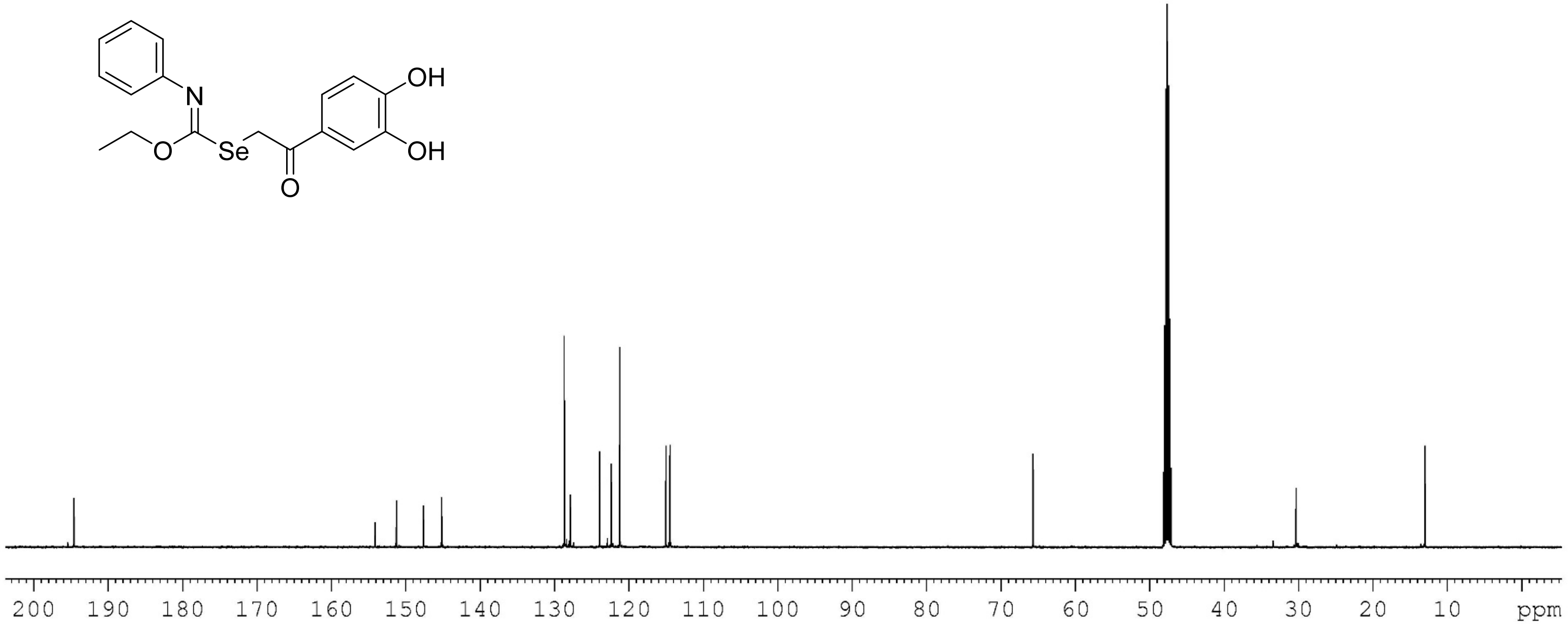

^{13}C-NMR (125.7 MHz, CD_3OD) of **18**

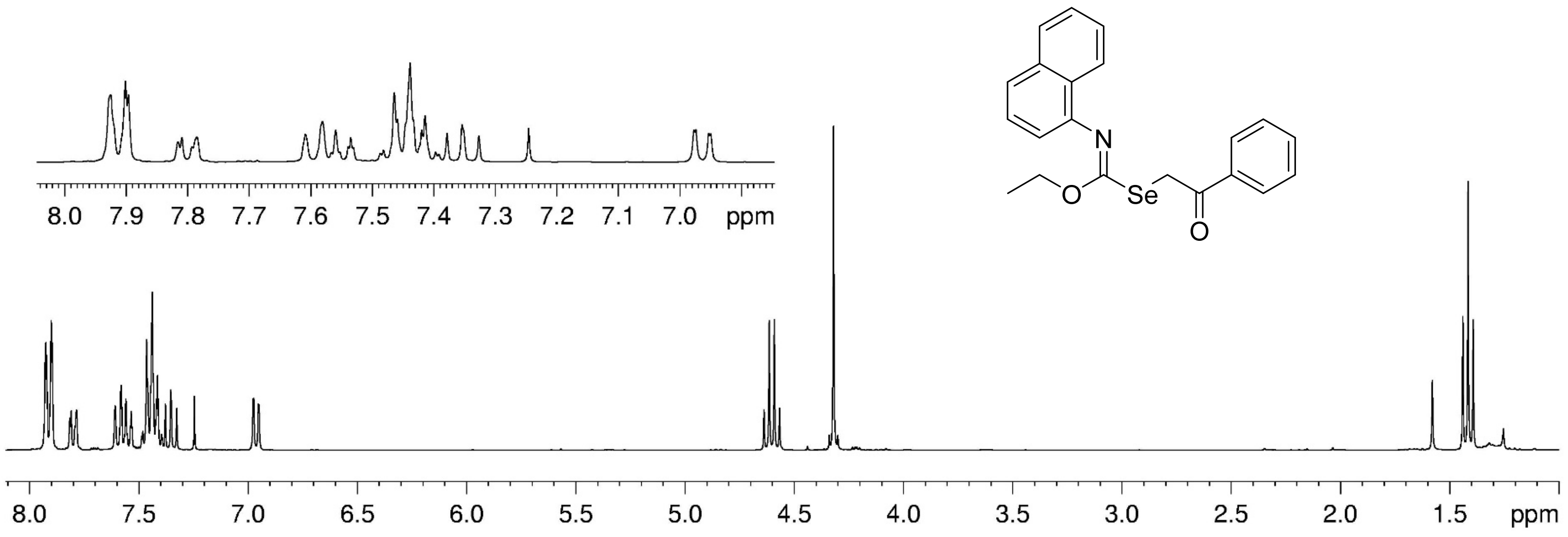

^{1}H-NMR (300 MHz, $CDCl_3$) of **19**

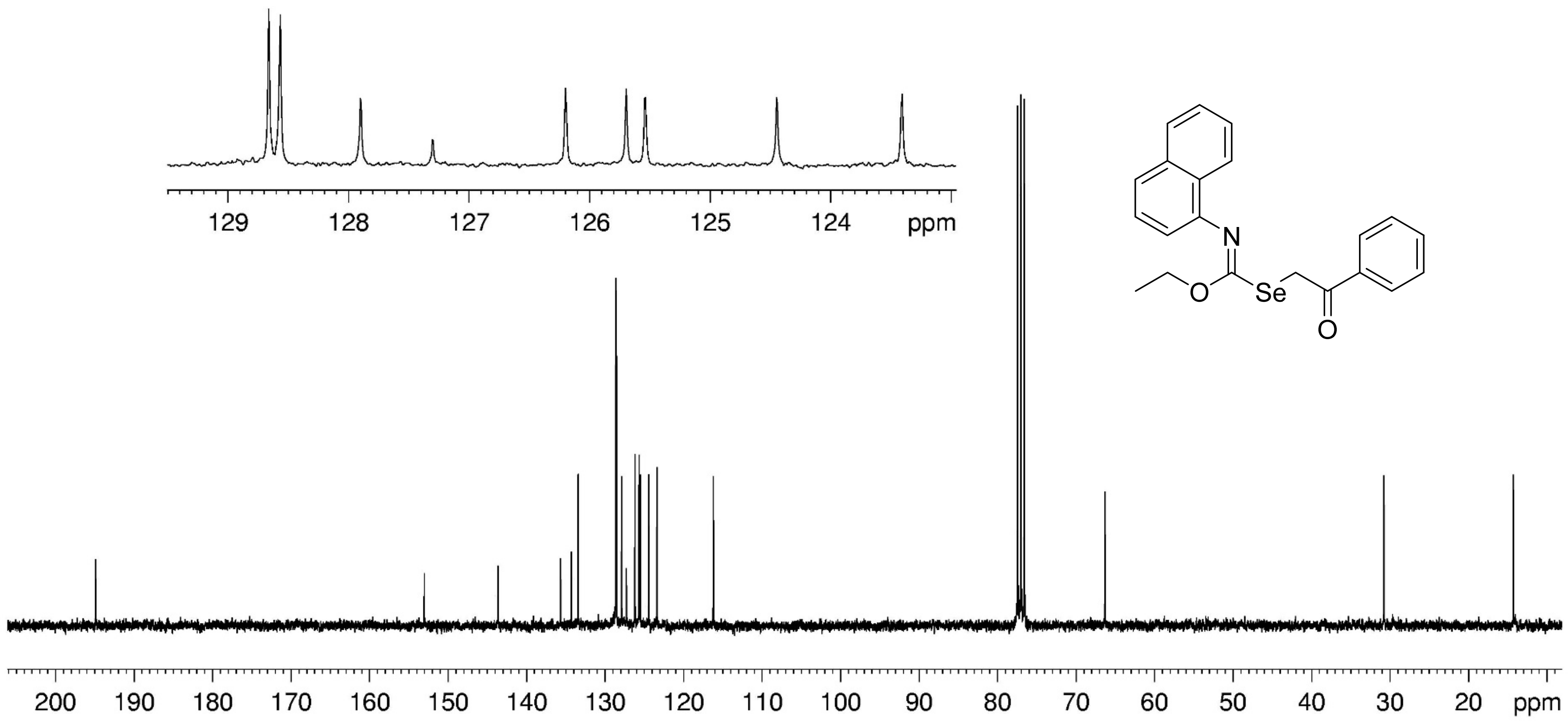

^{13}C-NMR (75.5 MHz, $CDCl_3$) of **19**

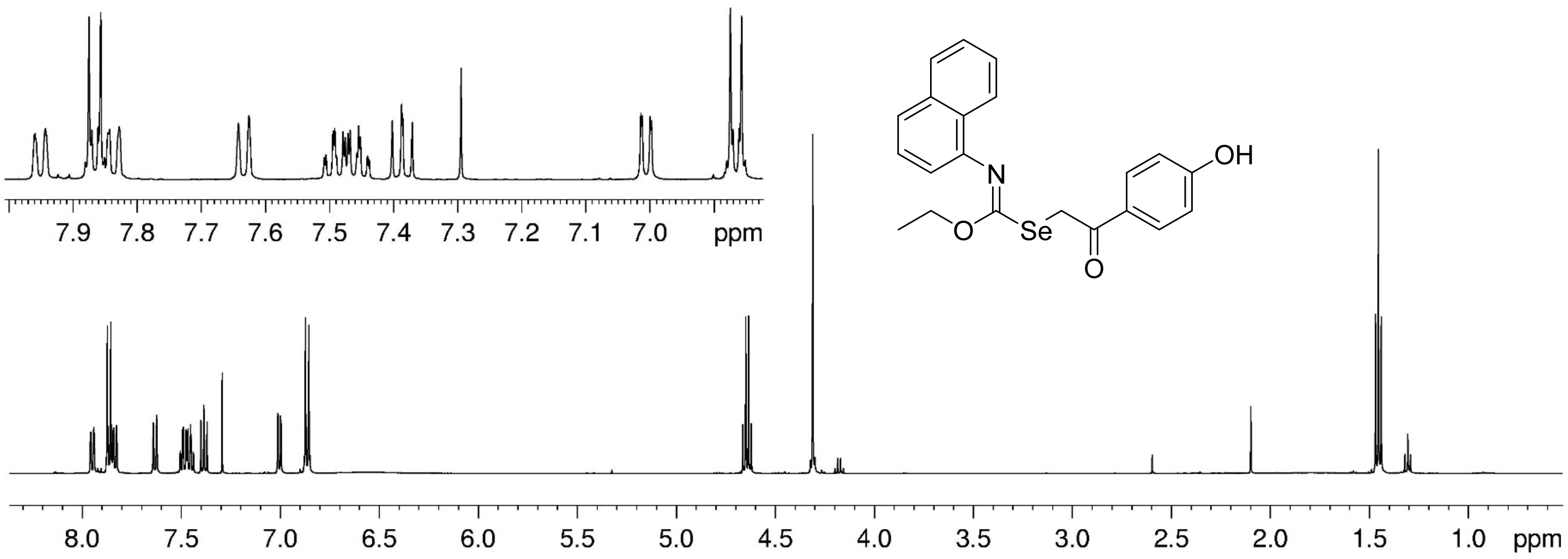

^{1}H-NMR (500 MHz, $CDCl_3$) of **20**

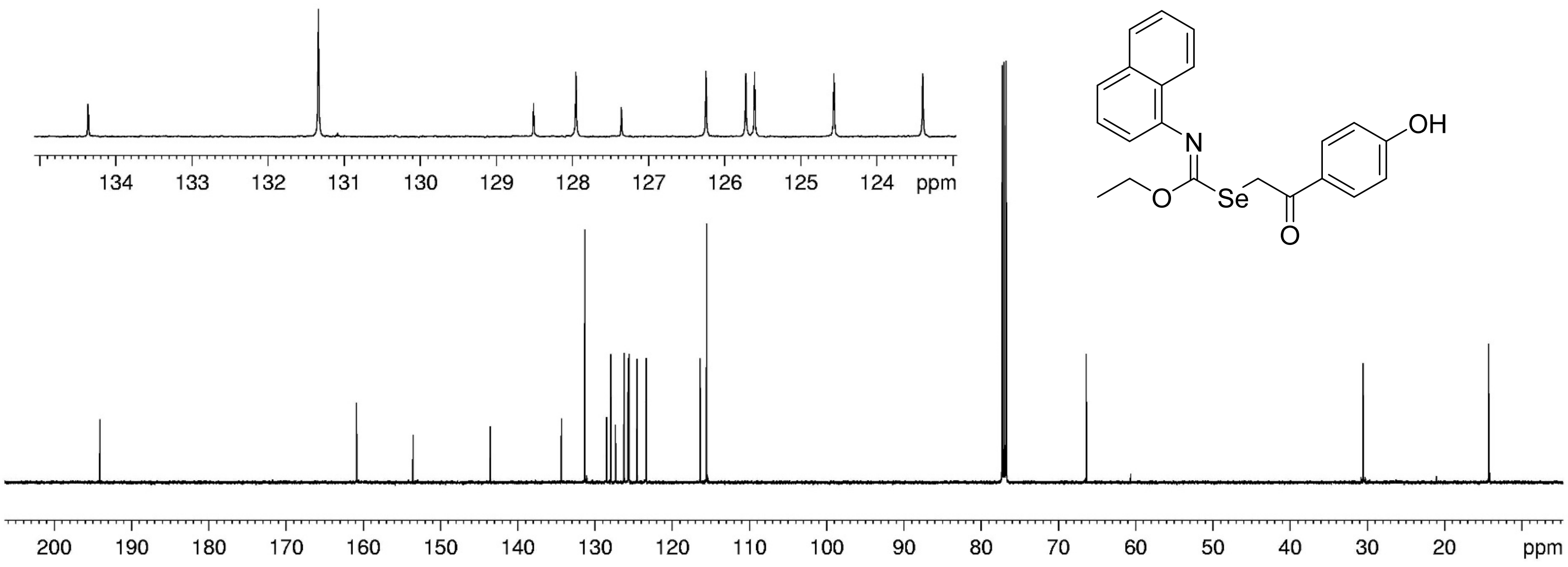

^{13}C-NMR (125.7 MHz, $CDCl_3$) of **20**

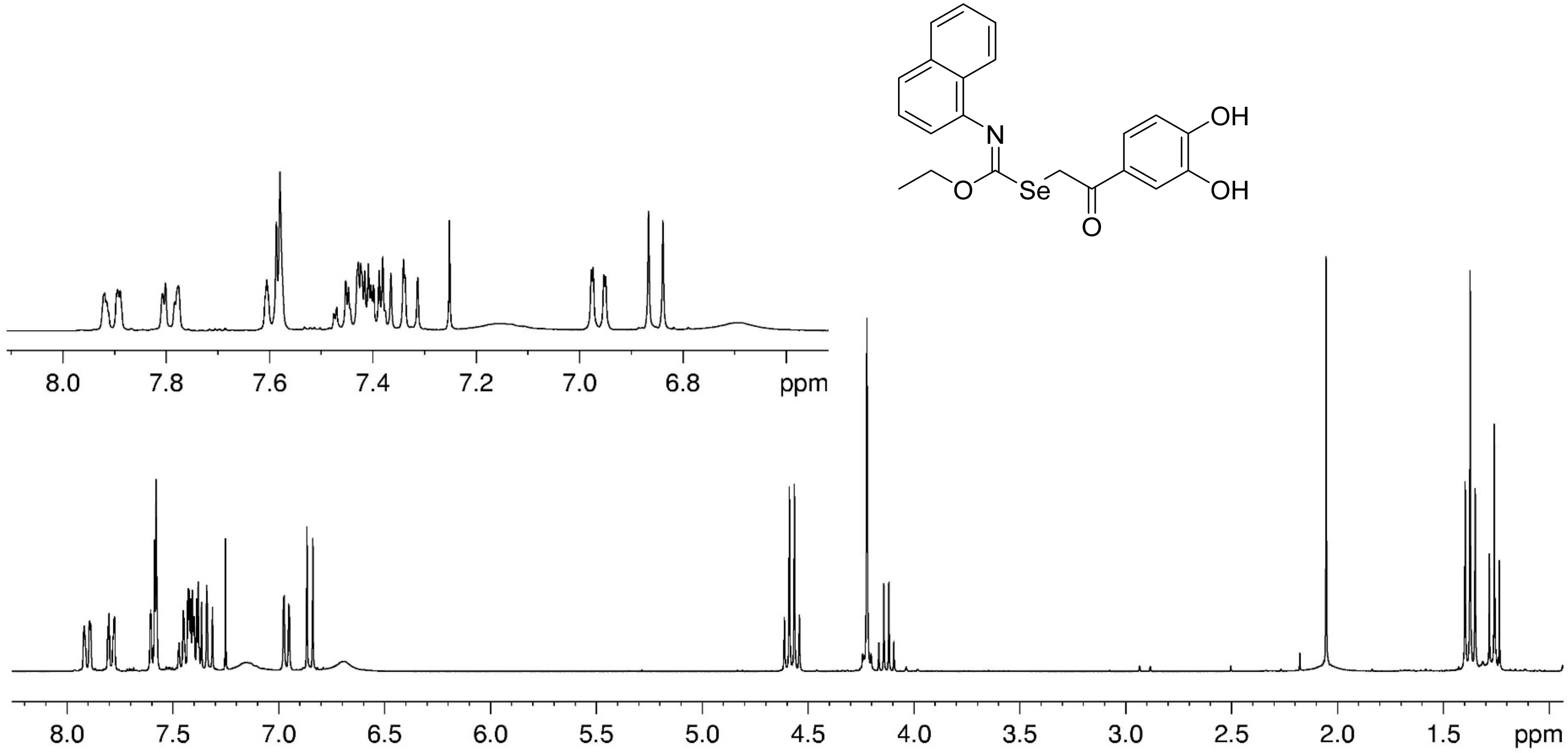

^{1}H-NMR (300 MHz, $CDCl_3$) of **21**

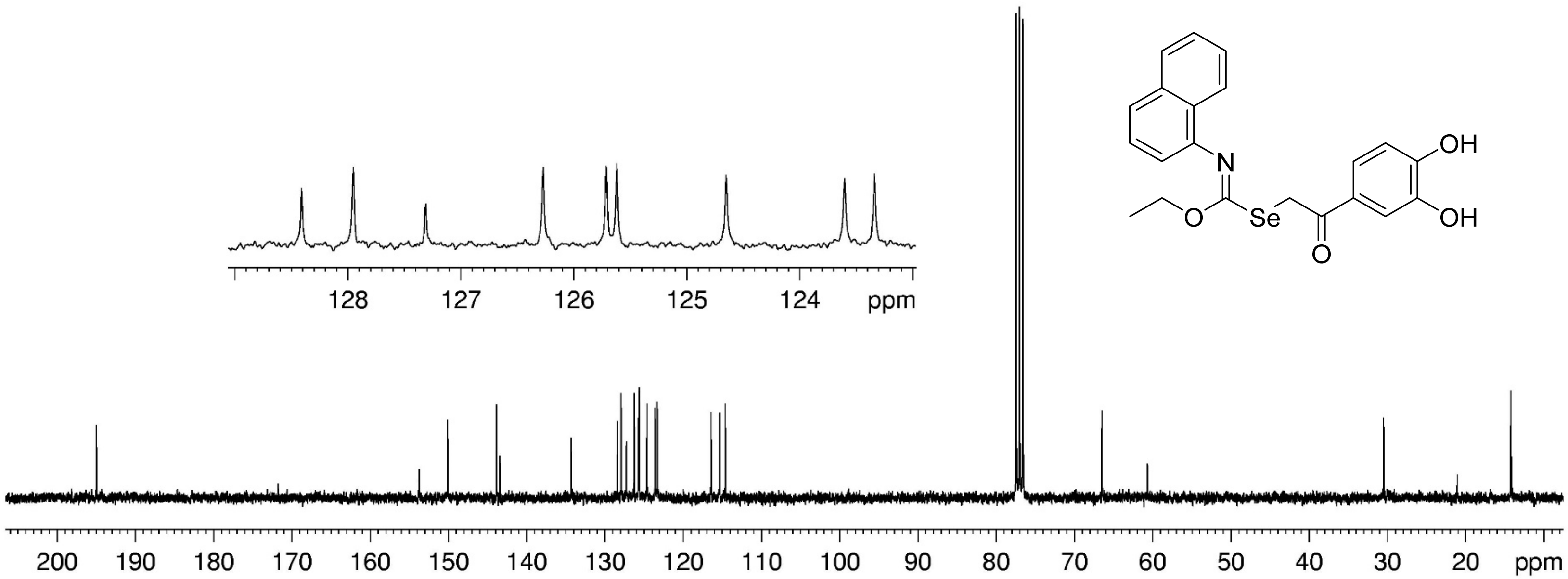

^{13}C-NMR (75.5 MHz, $CDCl_3$) of **21**

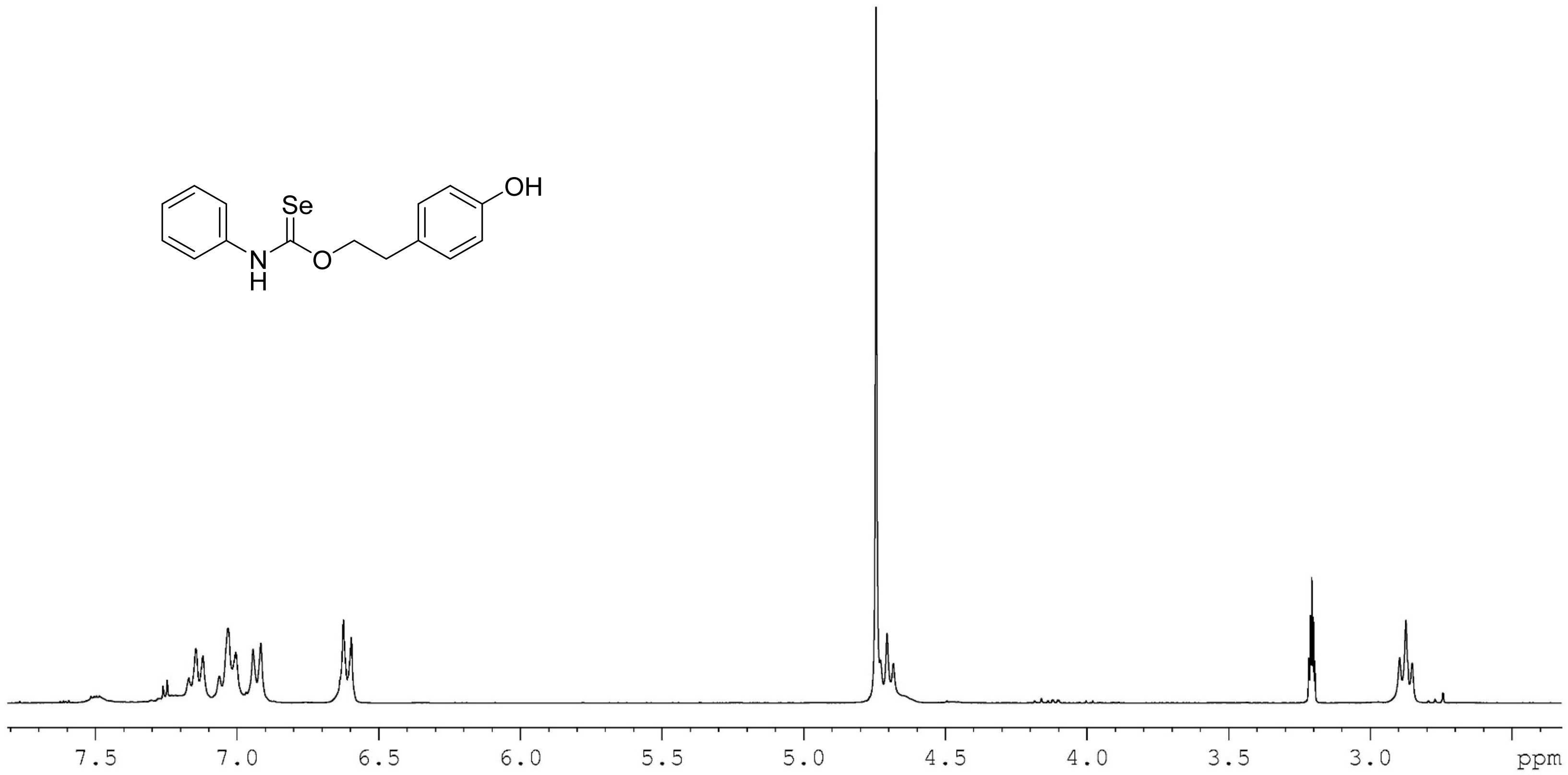

^{1}H-NMR (300 MHz, CD_3OD) of **24**

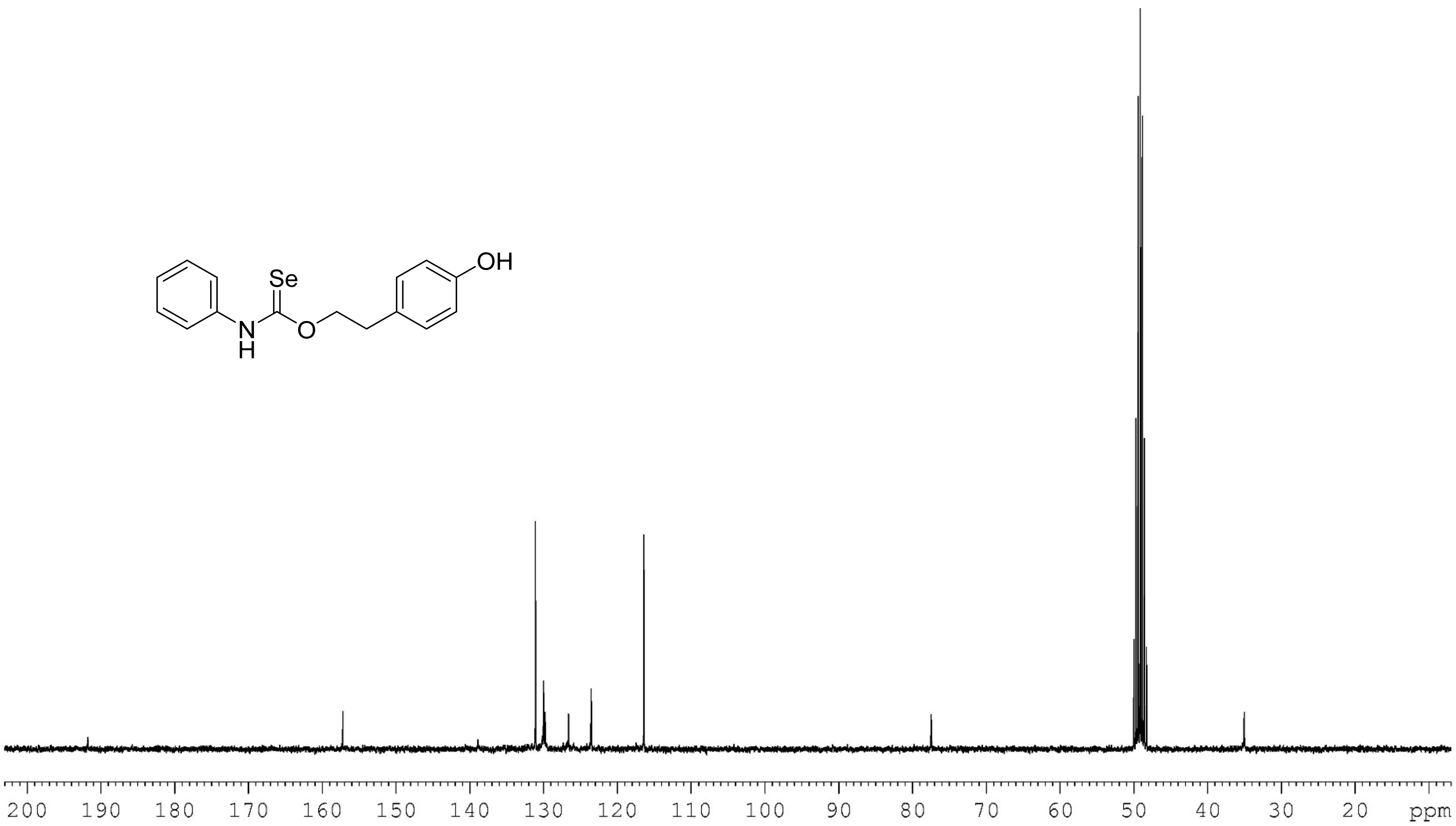

^{13}C-NMR (75.5 MHz, CD_3OD) of **24**

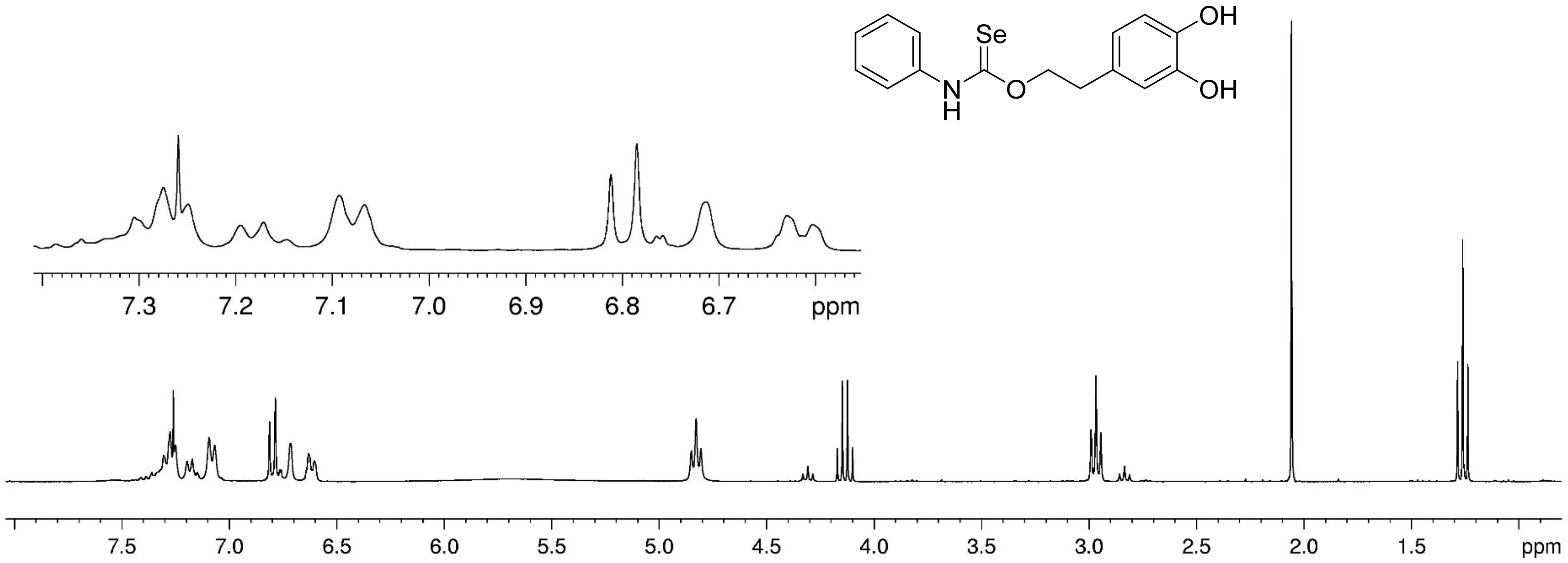

^{1}H-NMR (300 MHz, $CDCl_3$) of **25a**

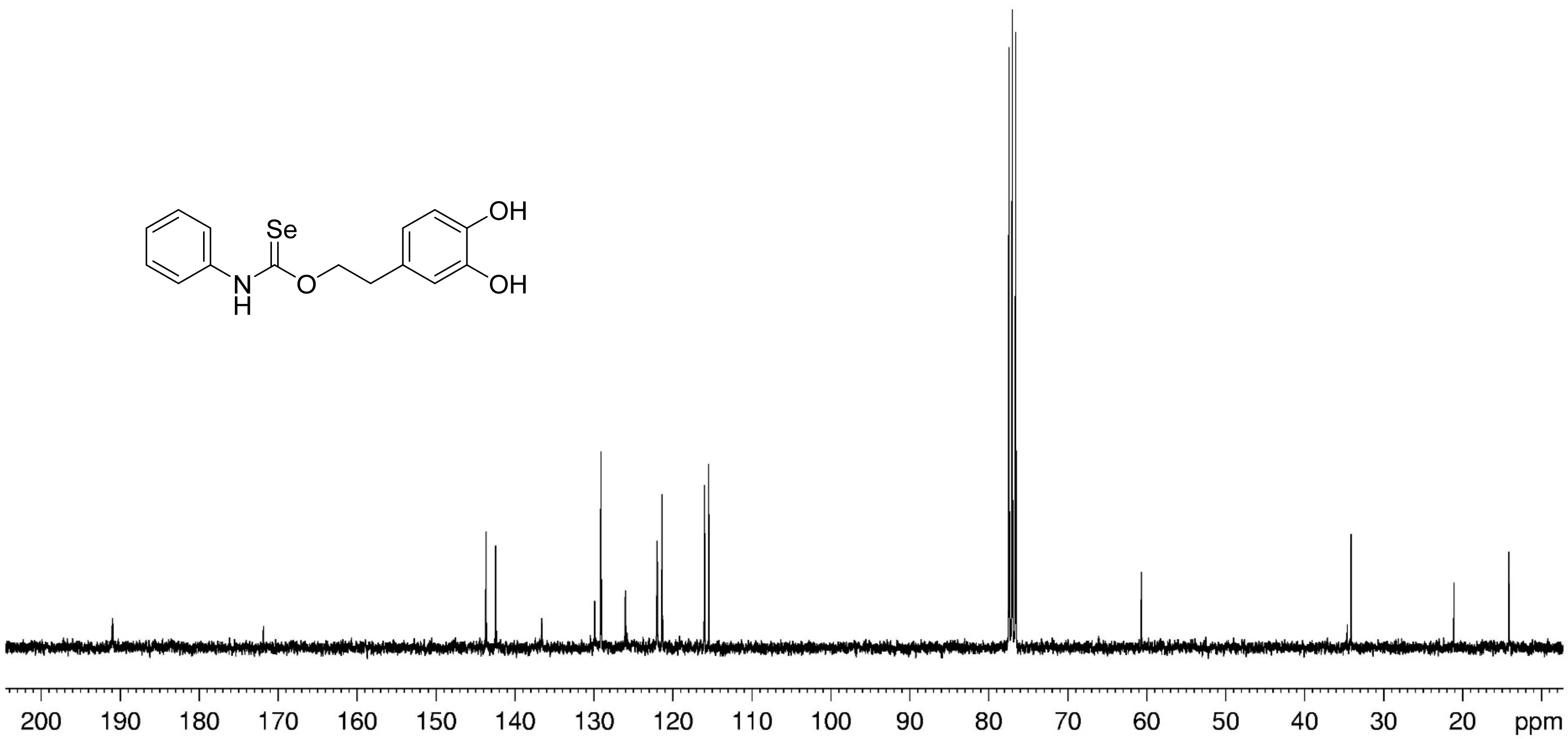

^{13}C-NMR (75.5 MHz, $CDCl_3$) of **25a**

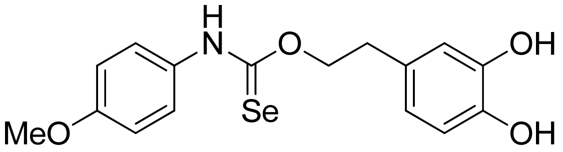

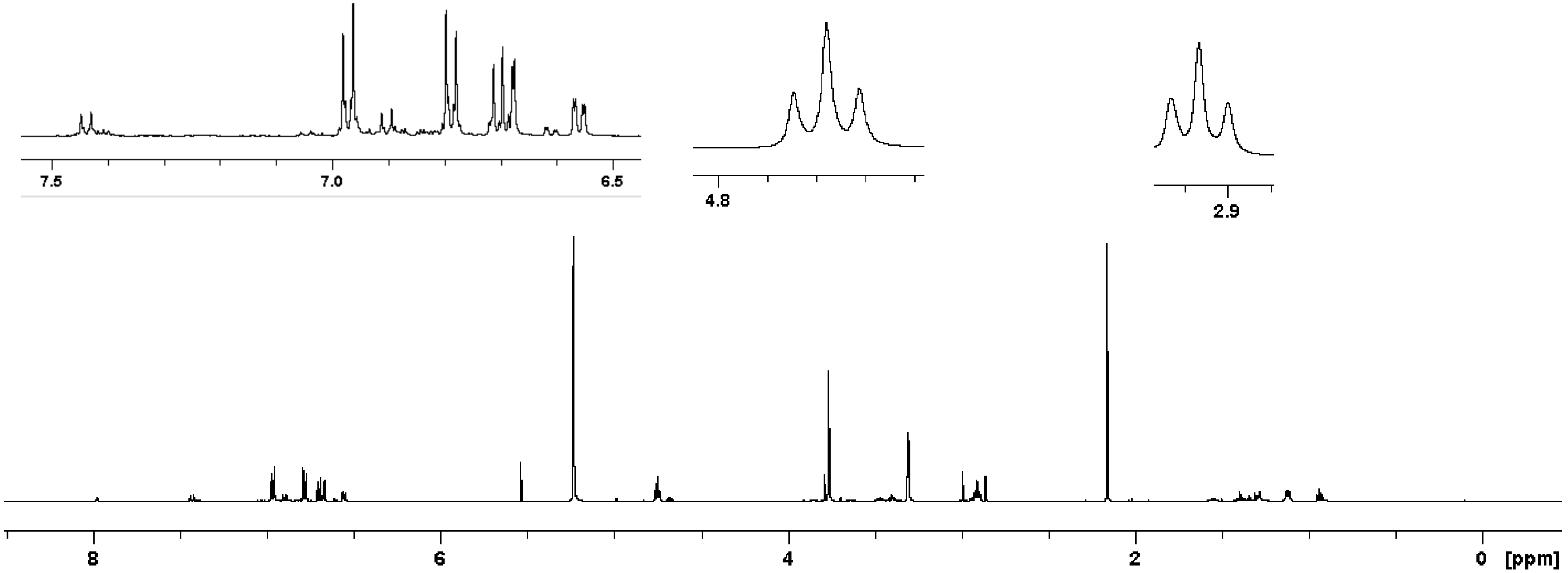

^{1}H-NMR (500 MHz, −20 ºC, CD_3OD) of **25b**

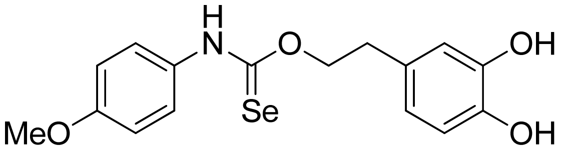

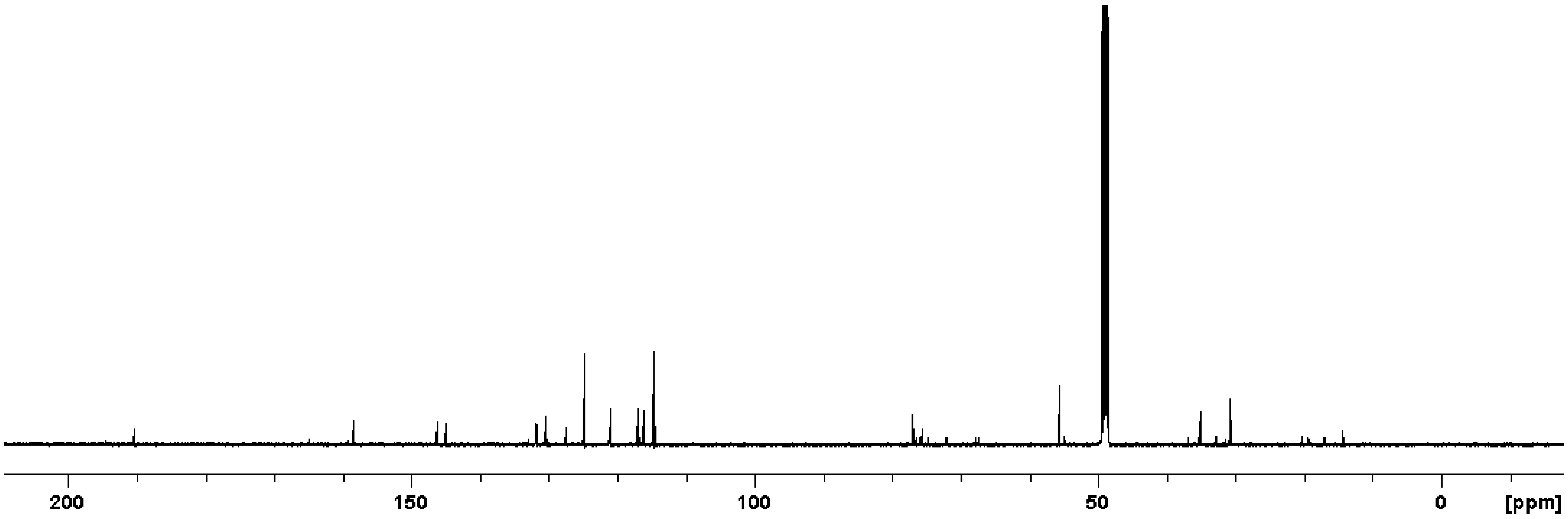

13C-NMR (125.7 MHz, −20 ºC, CD_3OD) of **25b**

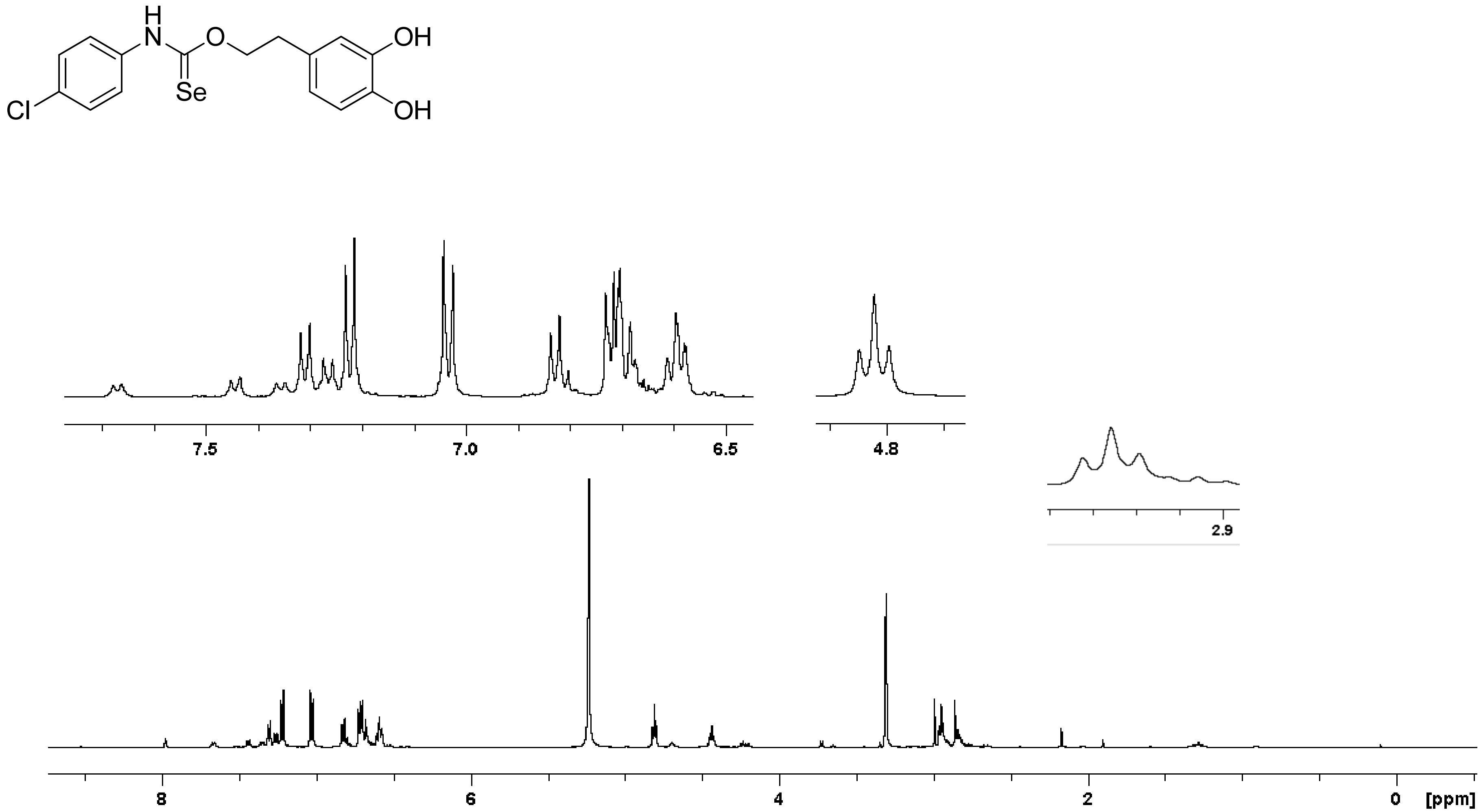

^{1}H-NMR (500 MHz, −20 ºC, CD_3OD) of **25c**

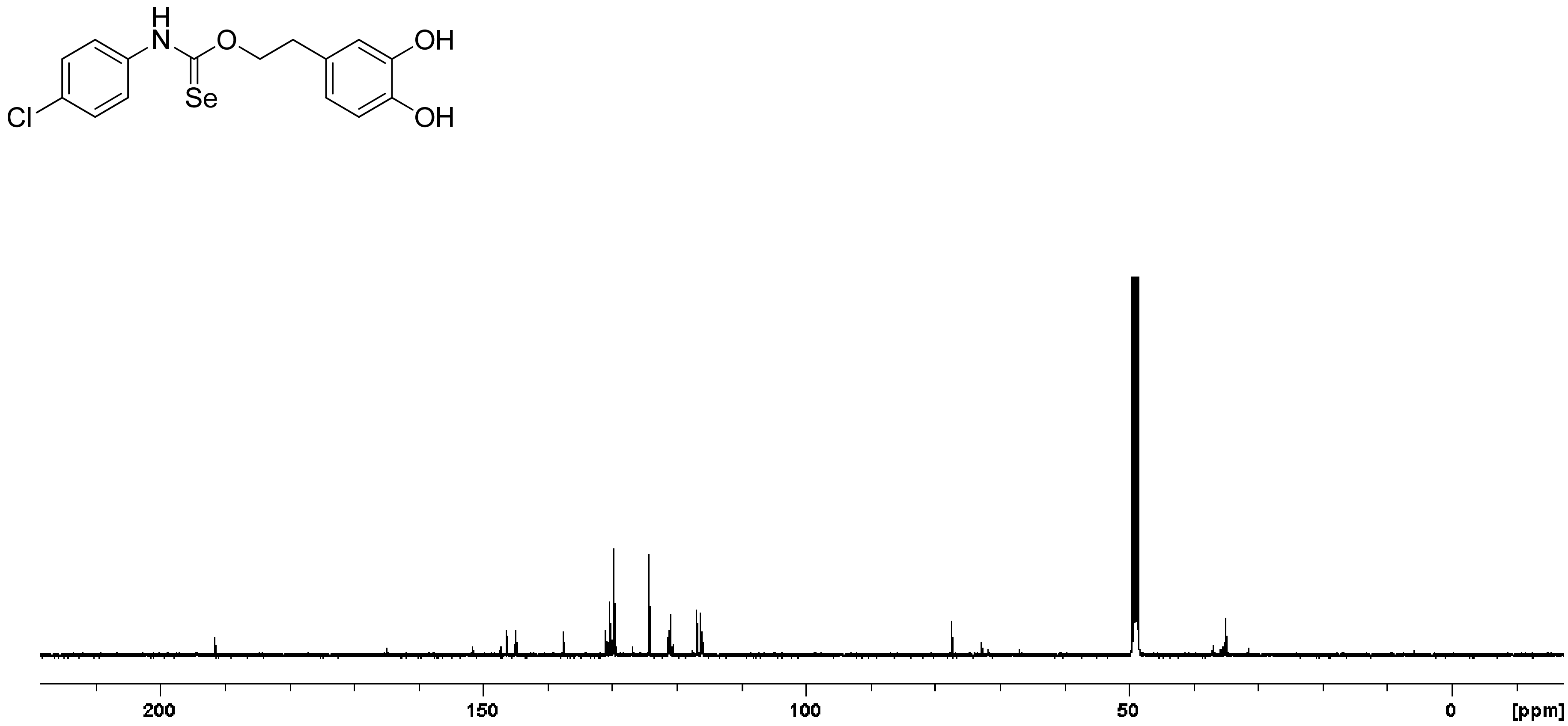

^{13}C-NMR (125.7 MHz, –20 ºC, CD_3OD) of **25c**

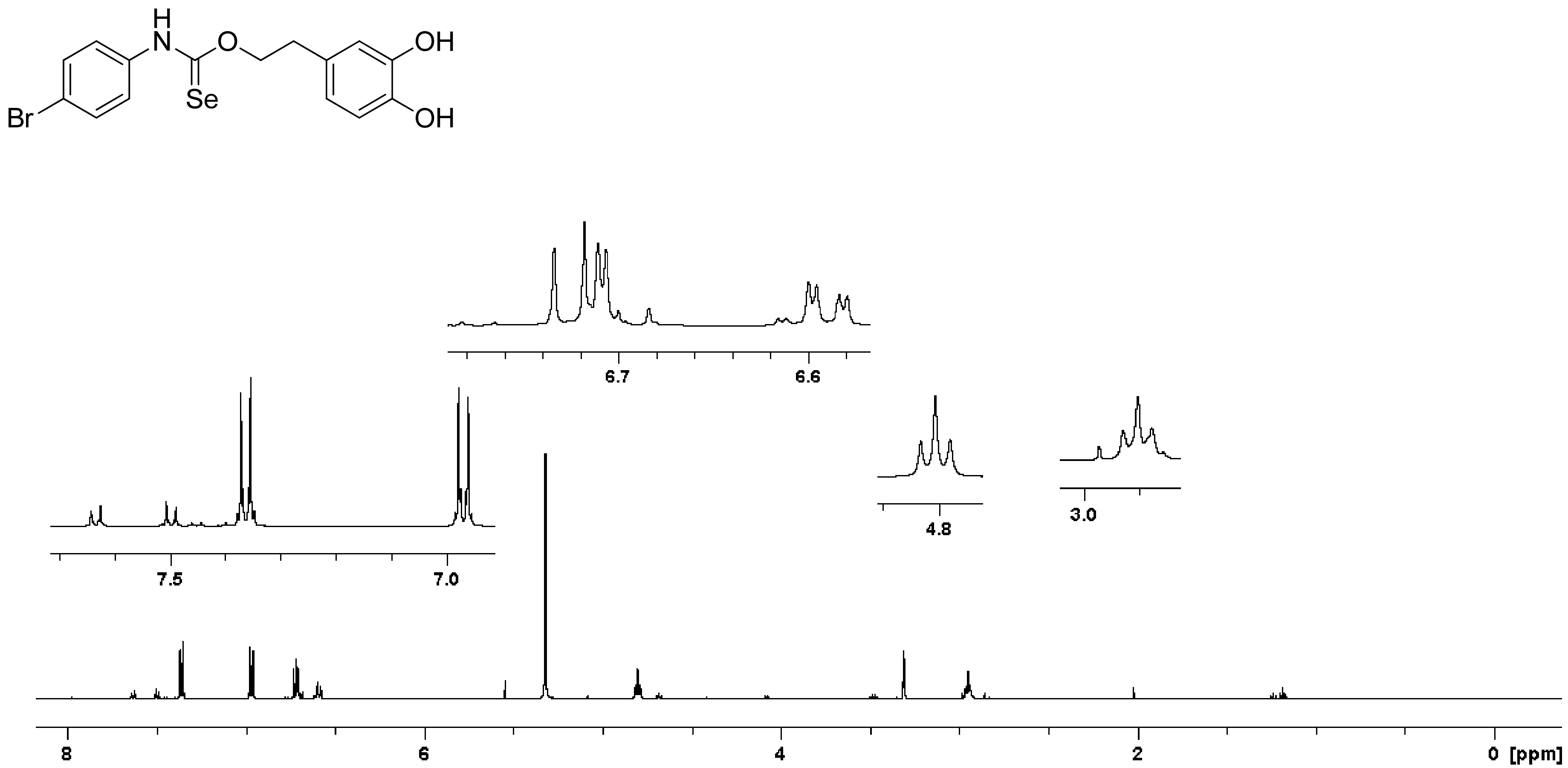

^{1}H-NMR (500 MHz, −20 ºC, CD_3OD) of **25d**

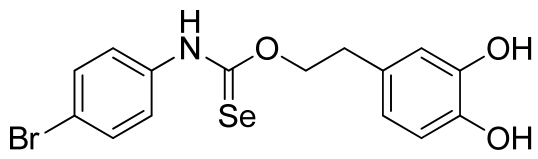

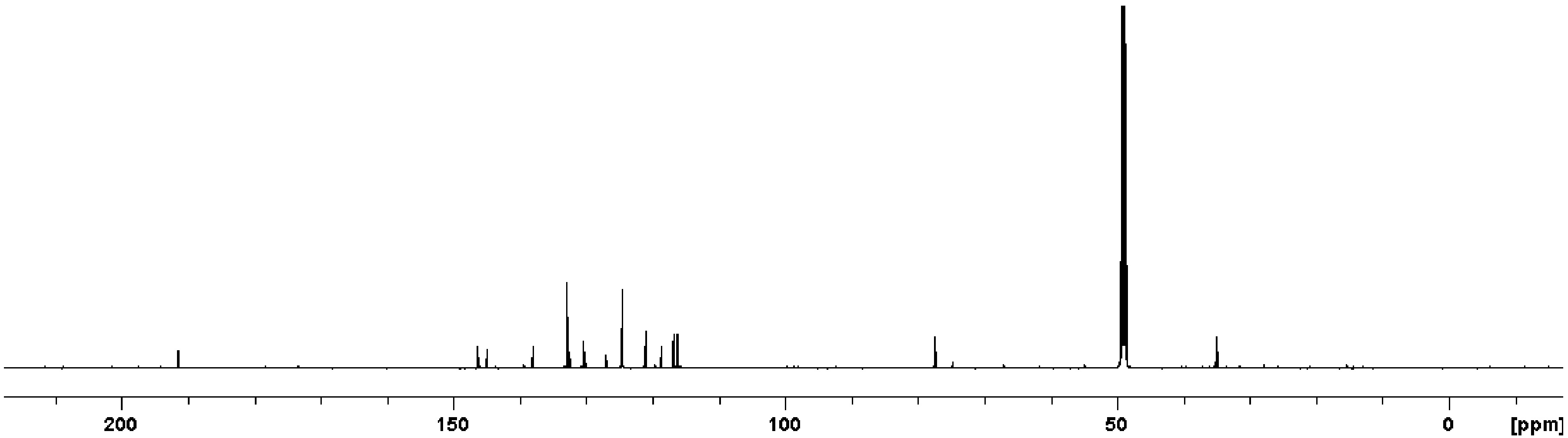

^{13}C-NMR (125.7 MHz, −20 ºC, CD_3OD) of **25d**

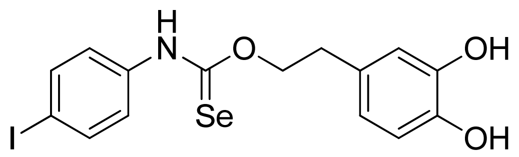

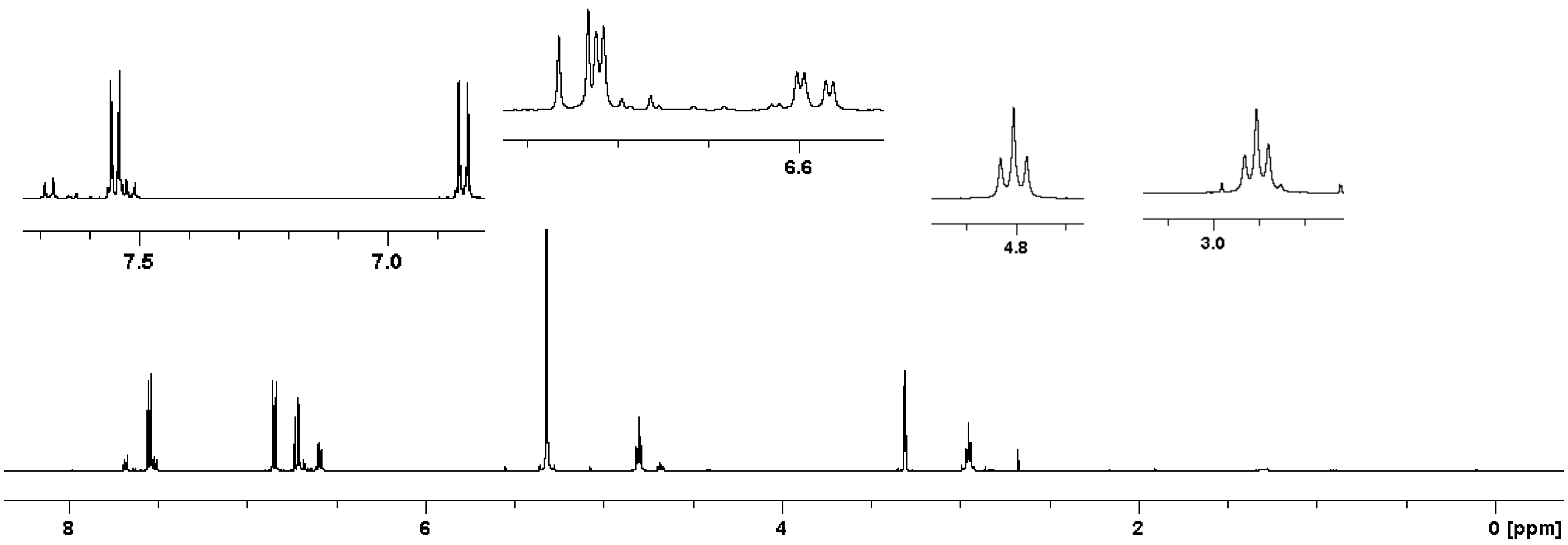

^{1}H-NMR (500 MHz, −20 ºC, CD_3OD) of **25e**

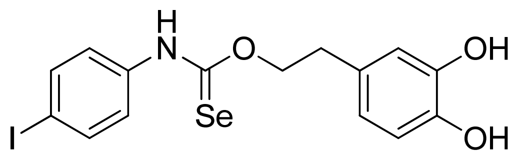

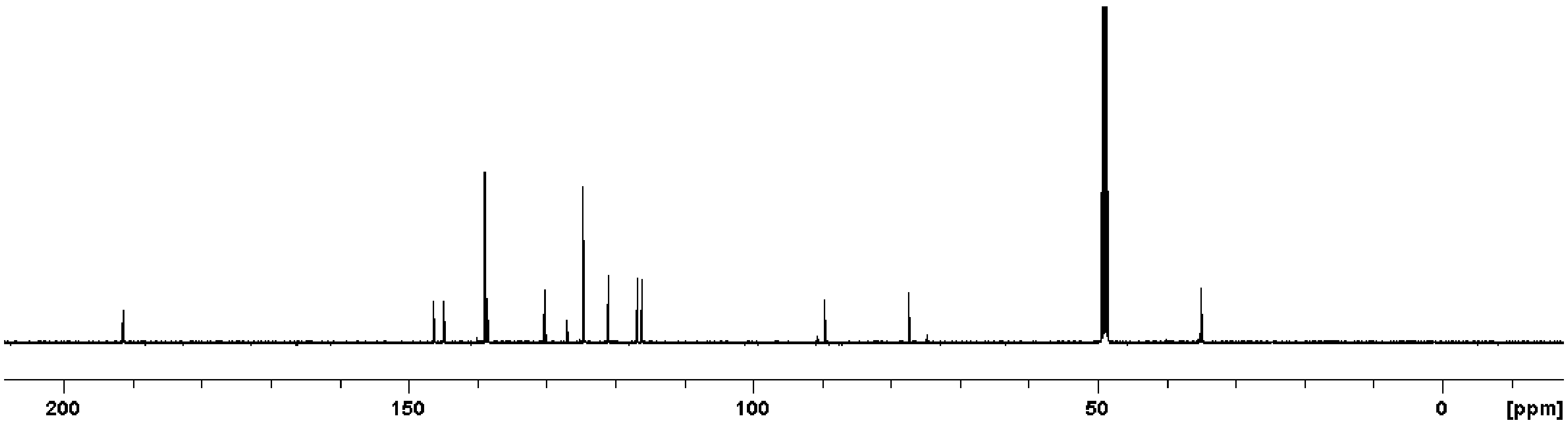

^{13}C-NMR (125.7 MHz, −20 ºC, CD_3OD) of **25e**

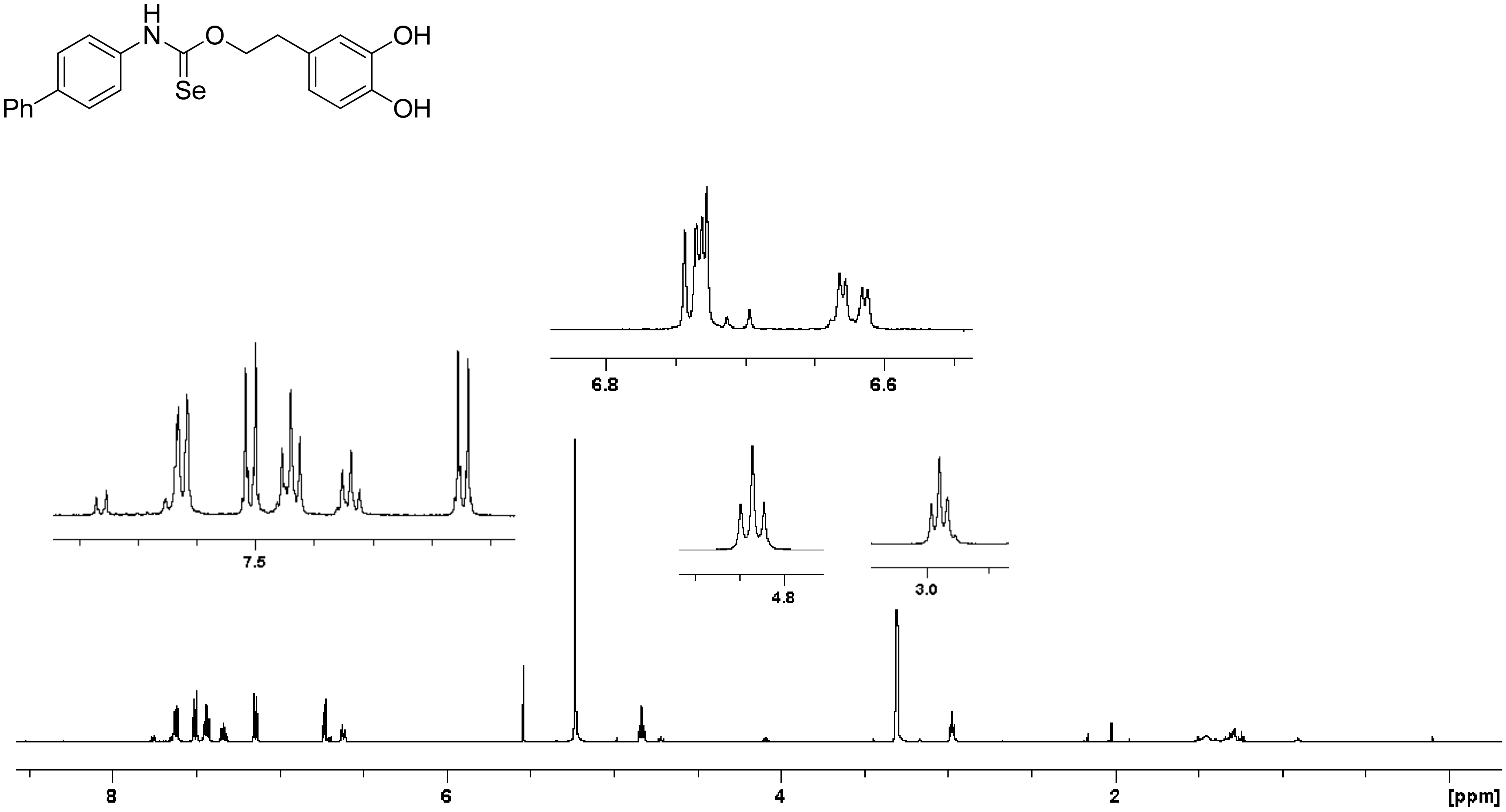

^{1}H-NMR (500 MHz, −20 ºC, CD_3OD) of **25f**

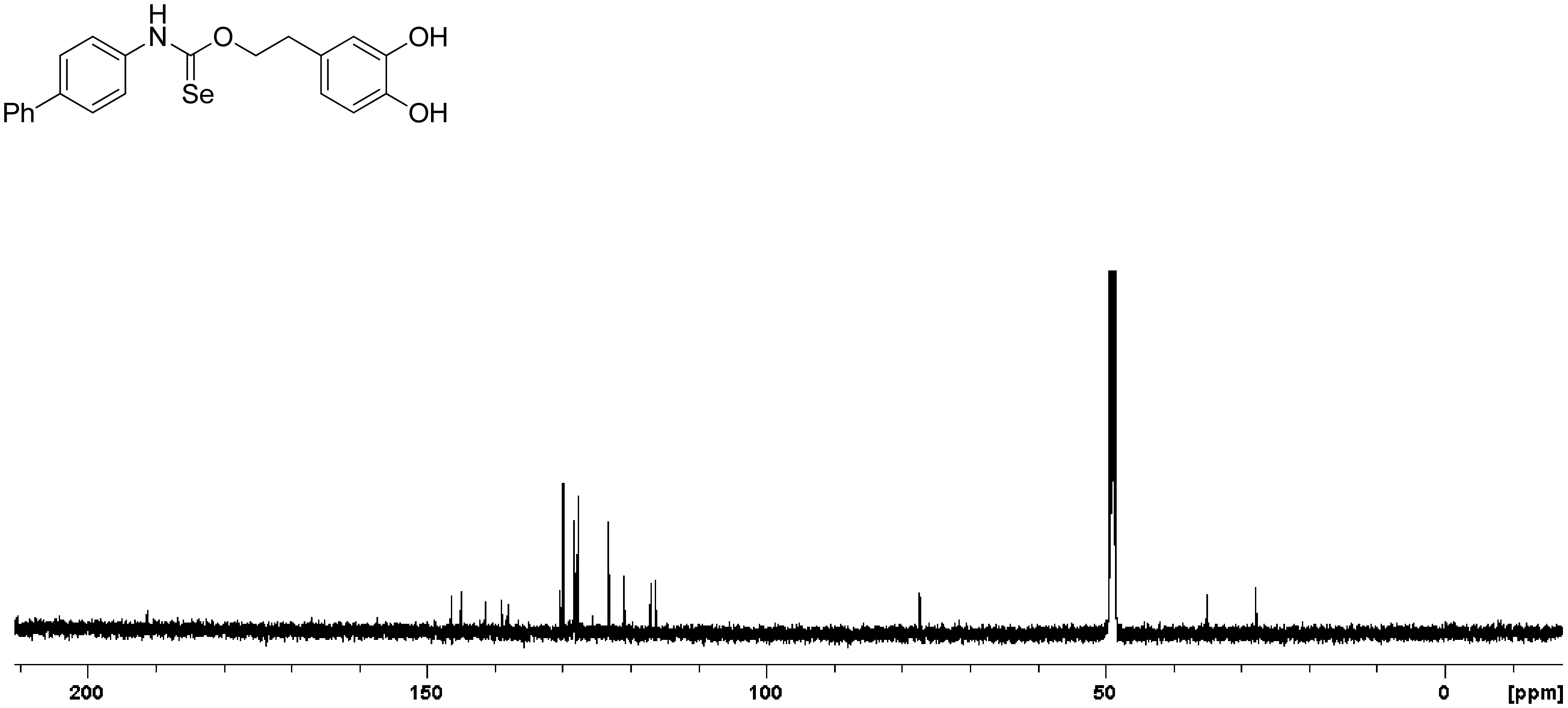

^{13}C-NMR (125.7 MHz, −20 ºC, CD_3OD) of **25f**

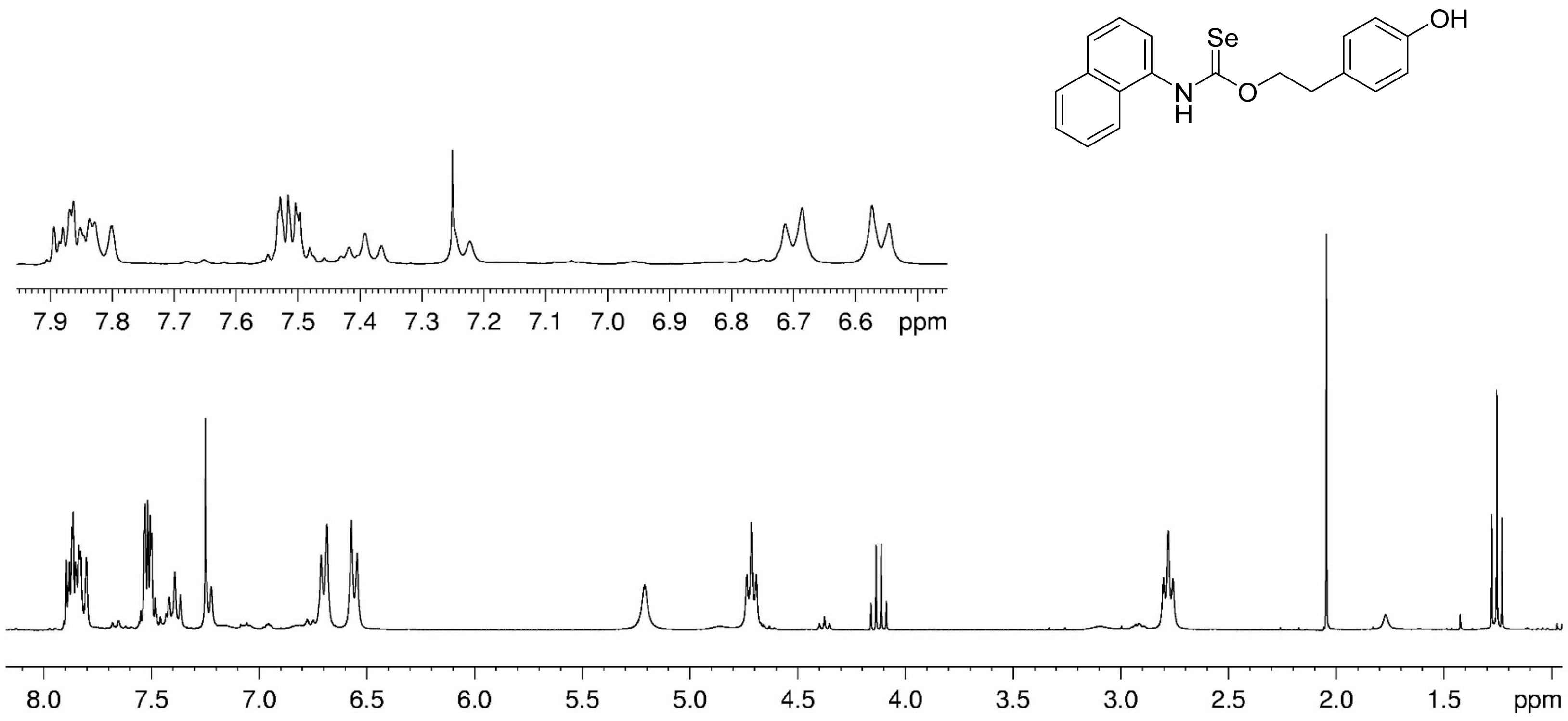

^{1}H-NMR (300 MHz, $CDCl_3$) of **26**

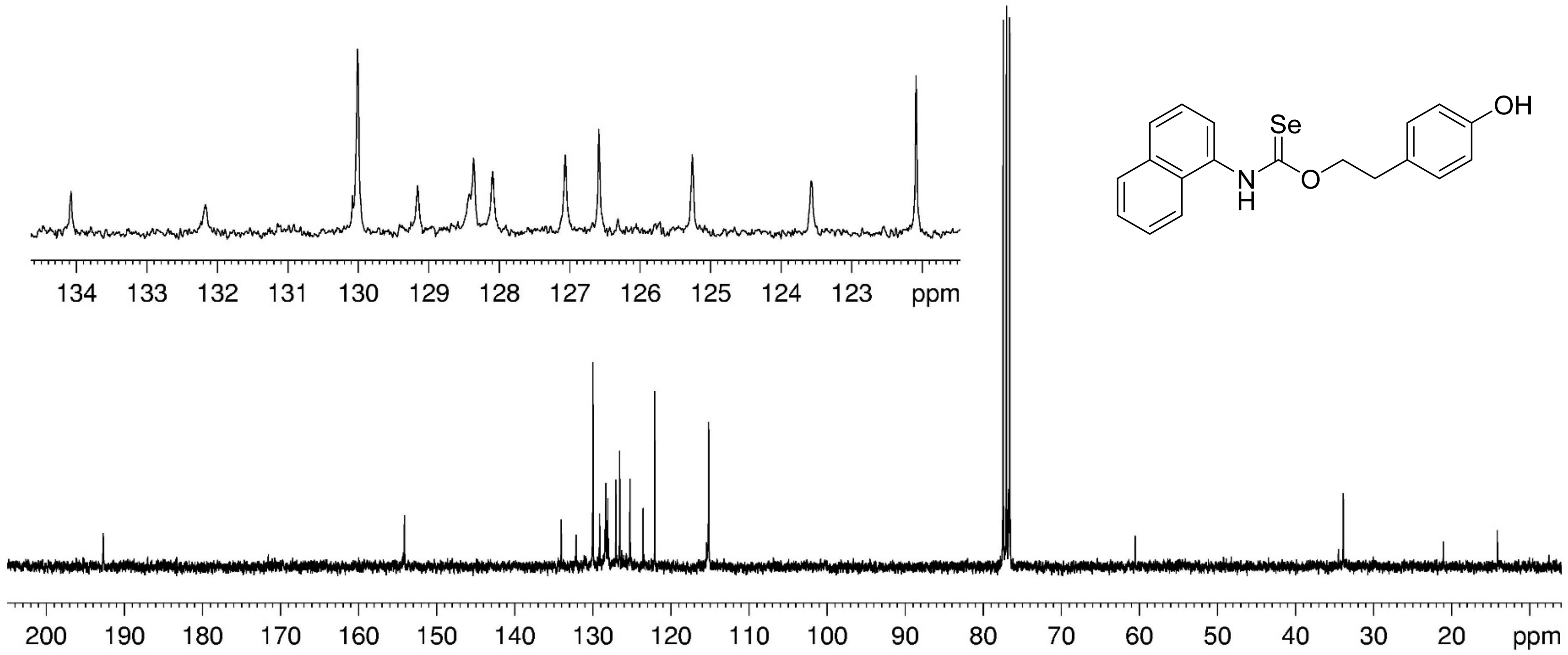

^{13}C-NMR (75.5 MHz, $CDCl_3$) of **26**

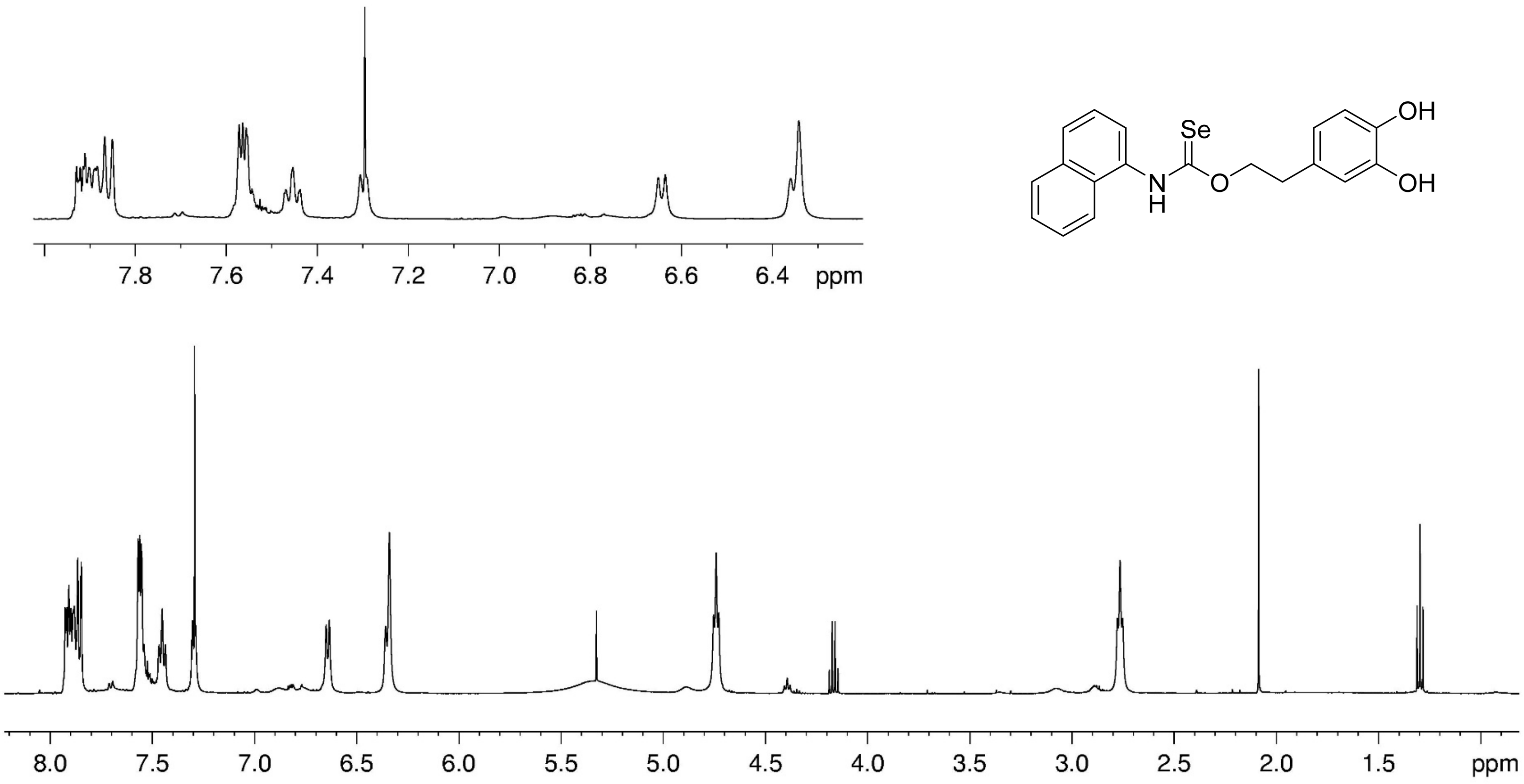

^{1}H-NMR (500 MHz, $CDCl_3$) of **27**

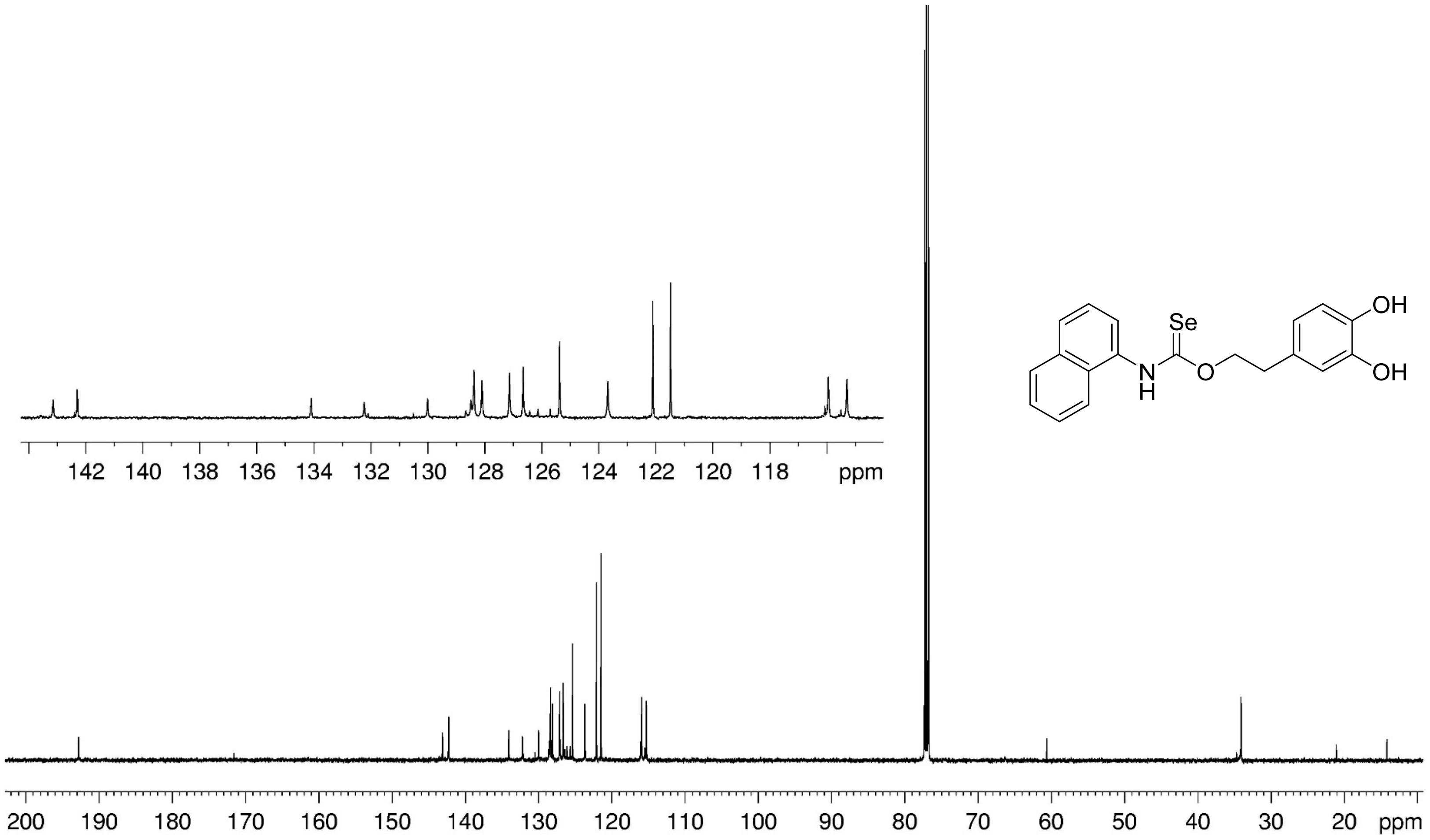

^{13}C-NMR (125.7 MHz, $CDCl_3$) of **27**
